# Effects of population aging on quality of life and disease burden: a population-based study

**DOI:** 10.1186/s41256-024-00393-8

**Published:** 2025-01-14

**Authors:** Jun-Yan Xi, Bo-Heng Liang, Wang-Jian Zhang, Bo Yan, Hang Dong, Yuan-Yuan Chen, Xiao Lin, Jing Gu, Yuan-Tao Hao

**Affiliations:** 1 Department of Medical Statistics, School of Public Health, Sun Yat-Sen University, 74Th Zhongshan 2Nd Rd, Yuexiu District, Guangdong, 510080 China; 2 Sun Yat-Sen Global Health Institute, Sun Yat-Sen University, Guangdong, 510080 China; 3 Center for Health Information Research, Sun Yat-Sen University, Guangzhou, 510080 China; 4 Department of Chronic Non-Communicable Disease Control and Prevention, Guangzhou Center for Disease Control and Prevention, Guangdong, 510440 China; 5 School of Health Sciences, Guangzhou Xinhua University, Guangdong, 510520 China; 6 Center for Public Health and Epidemic Preparedness and Response, Peking University, Haidian District, 38Th Xueyuan Road, Beijing, 100191 China; 7 Department of Epidemiology and Biostatistics, School of Public Health, Peking University, Beijing, 100191 China; 8 Key Laboratory of Epidemiology of Major Diseases (Peking University), Ministry of Education, Peking, 100191 China

**Keywords:** Attribution analysis, Burden of disease, Differential decomposition, Population aging, Prediction, Quality of life

## Abstract

**Background:**

As population aging intensifies, it becomes increasingly important to elucidate the casual relationship between aging and changes in population health. Therefore, our study proposed to develop a systematic attribution framework to comprehensively evaluate the health impacts of population aging.

**Methods:**

We used health-adjusted life expectancy (HALE) to measure quality of life and disability-adjusted life years (DALY) to quantify the burden of disease for the population of Guangzhou. The HALE and DALY projections were generated using both the Bayesian age-period-cohort models and the population prediction models. Changes in HALE and DALY between 2010–2020 and 2020–2030 were decomposed to isolate the effects of population aging. Three scenarios were analyzed  to examine the relative relationship between disease burden and population aging. In Scenarios 1 and 2, the disease burden rates in 2030 were assumed to  either remain at 2020 levels or follow historical trends. In Scenario 3, it was assumed that the absolute numbers of years of life lost (YLL) and years lived with disability (YLD) in 2030 would remain unchanged from the 2020 levels.

**Results:**

Between 2010 and 2020, 56.24% [69.73%] of the increase in male [female, values in brackets] HALE was attributable to the mortality effects in the population aged 60 and over, while − 3.74% [− 9.29%] was attributable to the disability effects. The increase in DALY caused by changes in age structure accounted for 72.01% [46.68%] of the total increase in DALY. From 2020 to 2030, 61.43% [69.05%] of the increase in HALE is projected to result from the mortality effects in the population aged 60 and over, while − 3.88% [4.73%] will be attributable to the disability effects. The increase in DALY due to changes in age structure is expected to account for 102.93% [100.99%] of the total increase in DALY. In Scenario 1, YLL are projected to increase by 45.0% [54.7%], and YLD by 31.8% [33.8%], compared to 2020. In Scenario 2, YLL in 2030 is expected to decrease by − 2.9% [− 1.3%], while YLD will increase by 12.7% [14.7%] compared to 2020. In Scenario 3, the expected YLL rates and YLD rates in 2030 would need to be reduced by 15.3% [15.4%] and 15.4% [15.6%], respectively, compared to 2020.

**Conclusions:**

The disability effects among the elderly population hinder improvements in quality of life, while changes in age structure are the primary driver of disease burden accumulation. To mitigate the excess disease burden caused by population aging, it is essential to achieve a reduction of more than 15% in the disease burden by 2030 compared to 2020. Our proposed attribution framework evaluates the health impacts of population aging across two dimensions: quality of life and disease burden. This framework enables comparisons of these effects over time and across different regions.

**Supplementary Information:**

The online version contains supplementary material available at 10.1186/s41256-024-00393-8.

## Background

Internationally, a country or region is typically classified as an aging society when 10% of its population is aged 60 and over, or 7% is aged 65 and over. The world is facing the complex challenges posed by an aging population. Since 1990, global life expectancy at birth has increased from 65.42 years to 73.52 years in 2019 [1]. Between 2020 and 2050, the global population aged 60 and over is expected to double to 2.1 billion, and the number of people aged 80 and over is expected to triple to 426 million [2]. As one of the world's most populous countries, China is rapidly becoming an aging society [3]. By 2030, it is estimated that the number of people aged 65 and over in mainland China will reach 258 million, accounting for 18.23 percent of the total population [4]. One of the consequences due to major demographic changes is an increase in the prevalence and incidence of age-related diseases. The elderly population has an increased susceptibility to many chronic and degenerative diseases, and the adverse health effects of these diseases often affect multiple organs or systems and accumulate with age, severely compromising the quality of life in old age [5, 6]. According to the Chinese Longitudinal Healthy Longevity Survey, disability in activities of daily living among older people in China increases with age, with disability rates of less than 5% for those aged 65–69, less than 20% for those aged 80–84, and about 40% for those aged 90–94 [7]. As older people are the most vulnerable group to disease and disability, and their demand for health services is higher than that of younger populations, the consistent evaluation on the health impacts of population aging is critical to ensuring the sustainability of modern societies [8].

Quantitatively attributing the health effects of population aging is crucial to addressing the challenges posed by an aging population. As populations age, the prevalence patterns of disease and injury shift significantly. Previous studies have demonstrated that the primary obstacles to effectively addressing this pattern of changes in the context of population aging, including misaligned global health priorities and the inadequate preparedeness of health systems to provide age-appropriate care for chronic disease [9]. Therefore, fully understanding and predicting these changes can provide important decision support for formulating public policies to actively respond to population aging. Health-adjusted life expectancy (HALE) and Disability-adjusted life years (DALY) are both comprehensive indicators used to measure population health by considering both mortality and morbidity. HALE adjusts life expectancy by accounting for year lived with disability from all diseases and injuries, offering a holistic measure of both quality and length of life [1]. In contrast, DALY quantifies the total number of healthy life years lost due to disease or injury, including years of life lost (YLL) and years lived with disability (YLD), thereby reflecting the overall disease burden [10]. While both indicators assess population health comprehensively, their perspectives differ. HALE emphasizes positive health outcomes, focusing on the duration of life spent in full health. Higher HALE values indicate better population health. DALY, on the other hand, highlights negative health impacts, measuring the epidemiological and socio-economic burden of disease and disability. Higher DALY values signify poorer population health.

Therefore, a comprehensive assessment of the health impact of population aging needs to combine the two dimensions: quality of life and burden of disease. However, an effective attribution strategy for analyzing the health impacts of population aging remains to be developed. The purpose of this study is to develop an attribution framework for a comprehensive assessment of the health impact of population aging from the perspective of the disease and injury spectrum, combining the two dimensions: quality of life and burden of disease using data from Guangdong Province of China as a case study.

## Methods

### Study design

**Fig. 1:**
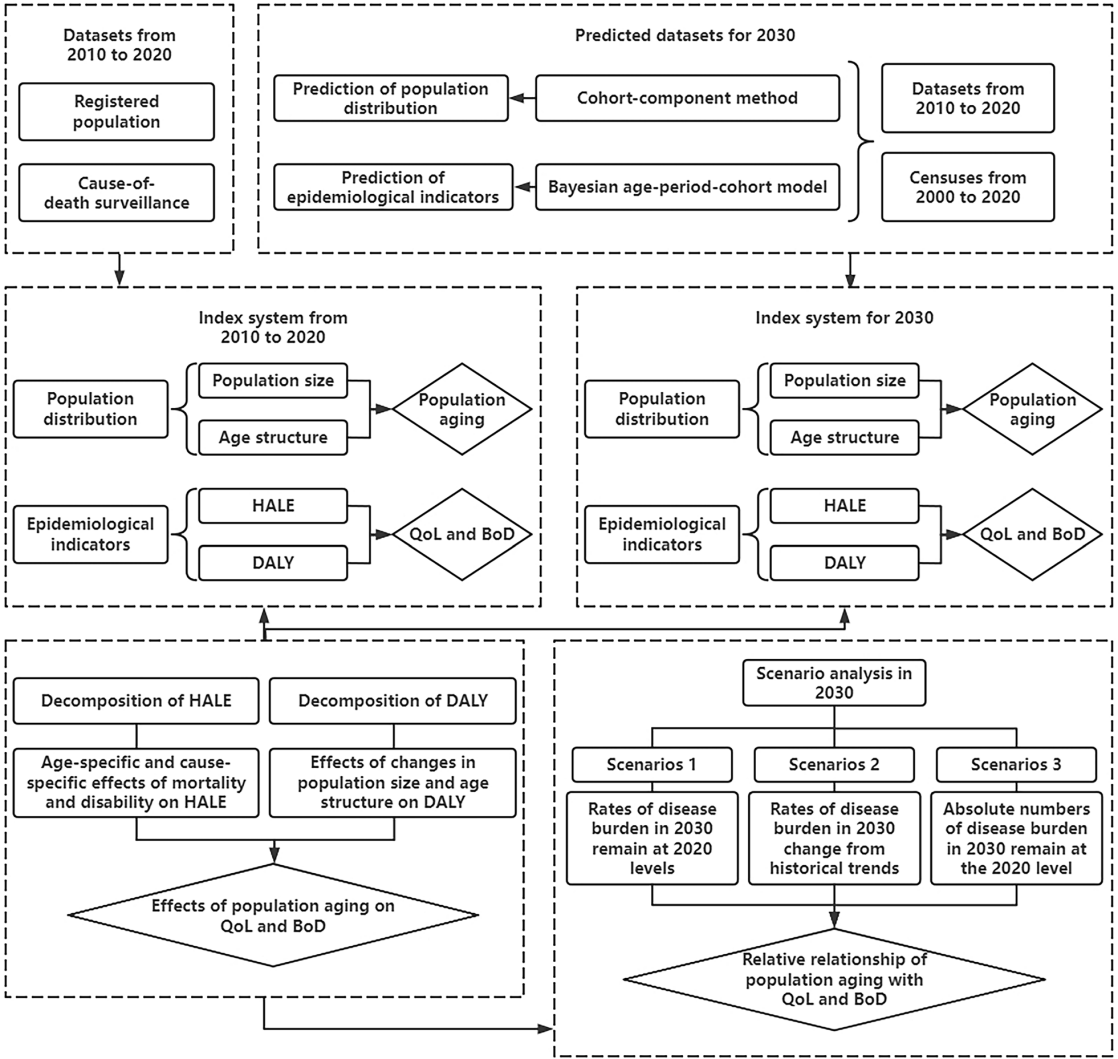
Flowchart of the attribution analysis framework in this study.* Note*: HALE = health-adjusted life expectancy; DALY = disability-adjusted life years; QoL = quality of life; BoD = burden of disease

We developed an attribution framework and applied it to the popualtion of Guangzhou, a representative developed megacity in Guangdong Province of China. This served asan example to provide an empirical basis for formulating health policies to implement more effective active health intervention strategies [11, 12]. The flowchart of our attribution framework is shown in Fig. 1 and detailed in online Appendix Table S1.

### Data source

The surveillance datasets on cause-of-death and the registered population of Guangzhou from 2010 to 2020 were obtained from the Guangzhou Centre for Disease Control and Prevention and the Public Security Bureau of Guangzhou Municipality. Details of these datasets have been reported in previous articles [13]. We classified the ICD-10 codes for causes of death according to the Global Burden of Disease Study (GBD) cause and sequelae hierarchy, including 3 categories of Level 2 causes and 21 categories of Level 3 causes [10]. For ICD-10 codes indicating unclear symptoms or signs as the underlying cause of death, we redistributed them according to the GBD garbage code list and previous research methods [10, 14]. (**online** **Appendix Text S1**) To predict the registered population of Guangzhou in 2030, we incorporated data on the age-specific fertility rate distribution of women aged 15 to 49, the birth rate, the sex ratio of newborns, and the age-specific migration rate distribution. These data were sourced from the Seventh National Population Census, the 1% National Population Sample Survey, and the Guangzhou Statistical Yearbook from 2000 to 2020. We also cited some results from the GBD, including YLL rates and YLD rates by sex, age, and cause in China from 2010 to 2019 [15]. The online Appendix Tables S2, S3, S4, and online Appendix Text S1 provide a mapping of ICD-10 to cause and sequelae, and garbage code in this study.

### Quality of life and disease burden measurement

We used HALE to measure quality of life and DALY to quantify the burden of disease. YLL and YLD by age are the main variables for HALE and DALY. YLL was calculated using the number of deaths in Guangzhou and the reference life table from the GBD, while YLD was determined using the method proposed by the WHO [16]. DALY is defined as the sum of YLL and YLD. HALE accounts for the loss of healthy lifespan due to death or disability across all ages. It was calculated using the abbreviated life table and the Sullivan method. The online Appendix Text S2 provides details of the methods used to calculate HALE and DALY.

To predict HALE and DALY in 2030, epidemiological indicators (mortality, YLL rates, and YLD rates) and population projections were required. Epidemiological indicators for 2030 were estimated using the Bayesian age-period-cohort (BAPC) model, while population distribution for 2030 was obtained through a population prediction model based on the cohort-component method. The BAPC model examines the main effects of three factors—age, period, and birth cohort—on changing trends in rates and predicts future changes. In our study, the BAPC model used a second-order random walk prior, which assumes that period effects evolve gradually over time rather than being driven by random effects [17]. The population prediction model based on the cohort-component method divides the population into cohorts by age and iteratively forecasts the number of individuals in each cohort according to demographic change patterns and the forecast cycle [4, 18]. This study took into account the policy direction of China, the current development context of Guangzhou, and insights from previous studies to set the parameters of population change, including three projections scenarios: high, medium, and low population development. The online Appendix Texts S3 and S4 provide methodological details on the projections of epidemiological indicators and population.

### Attribution analysis of the impact of population aging on quality of life

To examine the impact of population aging on quality of life during the periods 2010 to 2020 and 2020 to 2030, we decomposed the changes in HALE into the contributions from different causes. We defined the attributable contributions of people aged 60 and over as the health effects of population aging. The decomposition method for changes in HALE was based on the Sullivan and Arriaga method and consisted of two steps [19]. First, HALE changes were decomposed into age-specific mortality and disability effects. The mortality effects refer to the change in HALE when death changes while disability remains unchanged. The disability effects refer to the change in HALE when only disability changes while death remains the same. The mortality and disability effects were then further decomposed into the contributions of different causes. Detailed mathematical formulae are given in the online Appendix Text S5.

### Attribution analysis of the impact of population aging on disease burden

To examine the impact of population aging on the burden of disease from 2010 to 2020 and 2020 to 2030, we attributed the differences in DALY to the contributions of population age structure, population size, and all other factors. We defined DALY attributable to population age structure as the health effects of population aging. The decomposition method for changes in DALY assumed that one of the three factors changed between the first and second time points, while the others did not change. The expected DALY at the second time point was then calculated based on this assumption and compared with the DALY at the first time point to attribute the changes in DALY caused by specific factors [20]. Detailed mathematical formulae are given in the online Appendix Text S6.

### Scenario analysis

Three scenarios were developed to analyze the relative relationship between changes in the burden of premature death (YLL rates) and disability (YLD rates) and population changes. Scenarios 1 and 2 explore the influence of population changes on the burden of disease by assuming that YLL and YLD rates in 2030 remain at 2020 levels or follow historical trends (modeling projections). Scenario 3 assumes that the health effects of population changes are neutralized by 2030, maintaining the absolute numbers of YLL and YLD at the 2020 levels. This scenario aims to achieve the expected burden of premature death and disability relative to the levels controlled in 2020. The mathematical expressions of three scenarios are given in the online Appendix Text S7.

### Uncertainty analysis

We assumed that each input for the health measures and attribution models followed a triangular distribution with a minimum value $a$, a peak value $b$, and a maximum value $c$, where $a,b,c$ correspond to the lower, middle, and upper limits of the GBD estimates, respectively. Then, we generated 1000 draw-level estimates using the Monte Carlo method, assuming that the input parameters followed the triangular distribution [21]. 95% uncertainty intervals (UIs) were estimated using the 2.5th and 97.5th percentiles of the draws. The BAPC model estimated the observed rates and the predicted distribution between the 5th and 95th quantiles, using point estimates of the epidemiological indicators and the population as inputs. The population prediction model based on the cohort-component method set up three scenarios to show the possible development trend of the future population under different conditions, where the high and low scenarios defined the range of projection results and the middle scenario represented the most likely projection results [22].

## Results

### Characteristics of population aging from 2010 to 2030

**Fig. 2:**
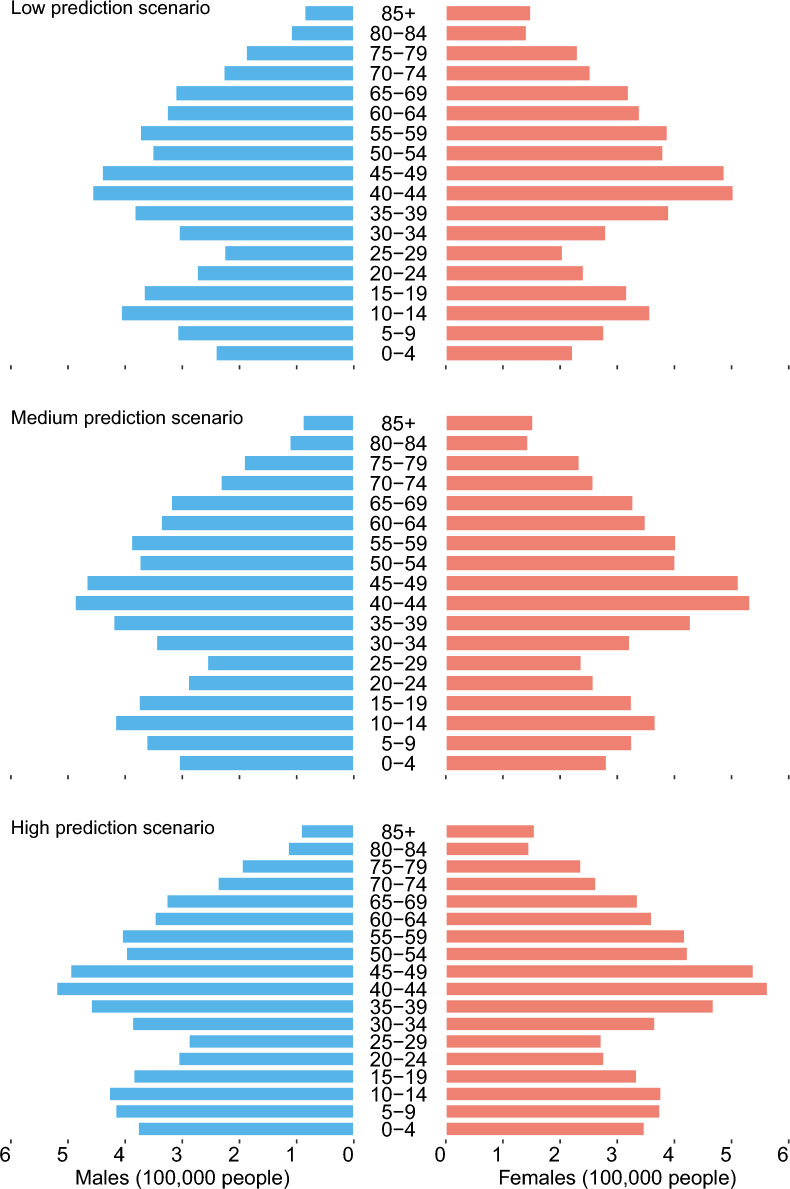
Population aging in 2030 under three prediction scenarios

Between 2010 and 2020, the proportion of people aged 60 and over in Guangzhou's total population increased from 14.5% to 18.2% (male: 13.4% to 17.2%, female: 15.5% to 19.3%). (online Appendix Figures S1) By 2030, population aging is expected to intensify. In the low scenario, the proportion of people aged 60 and over is projected to increase from 18.2% to 24.7% (male: 17.2% to 23.2%, female: 19.3% to 26.1%). In the medium scenario, the proportion is projected to reach 23.6% (male: 22.2%, female: 25.0%). In the high scenario, the proportion is projected to reach 22.6% (male: 21.3%, female: 23.9%). **(**Fig. 2**).**

### Decomposition of changes in health-adjusted life expectancy from 2010 to 2030

**Fig. 3:**
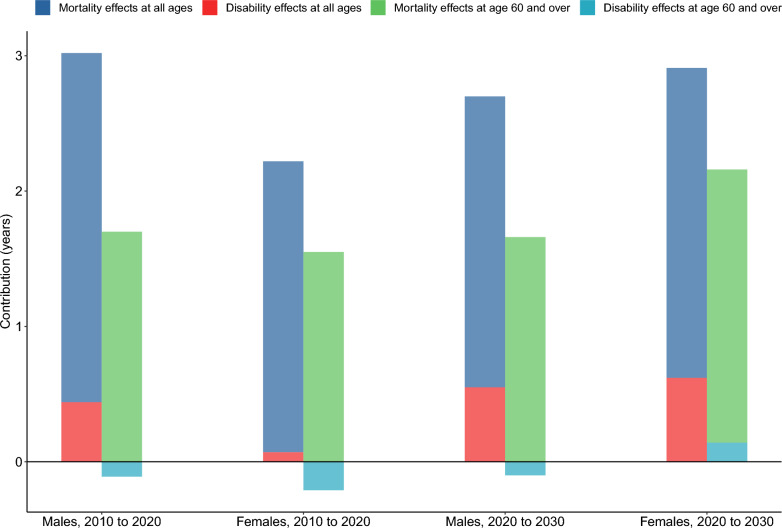
Decomposition of changes in health-adjusted life expectancy from 2010 to 2030

Between 2010 and 2020, 56.24% (1.70 years) of the increase in male HALE was attributed to the mortality effects among the population aged 60 and over, while − 3.74% (− 0.11 years) was attributed to the disability effects. For females, 69.73% (1.55 years) of the increase in HALE was due tothe mortality effects among those aged 60 and over, with − 9.29% (− 0.21 years) attributed to the disability effects. From 2020 to 2030, 61.43% (1.66 years) of the increase in male HALE is projected to be driven by the mortality effects among the population aged 60 and over, and − 3.88% (− 0.10 years) by the disability effects. For females, 69.05% (2.02 years) of the increase in HALE is expected to result from the mortality effects among those aged 60 and over, with 4.73% (0.14 years) attributed to the disability effects. **(**Fig. 3** and** online Appendix Figure S2).

**Fig. 4:**
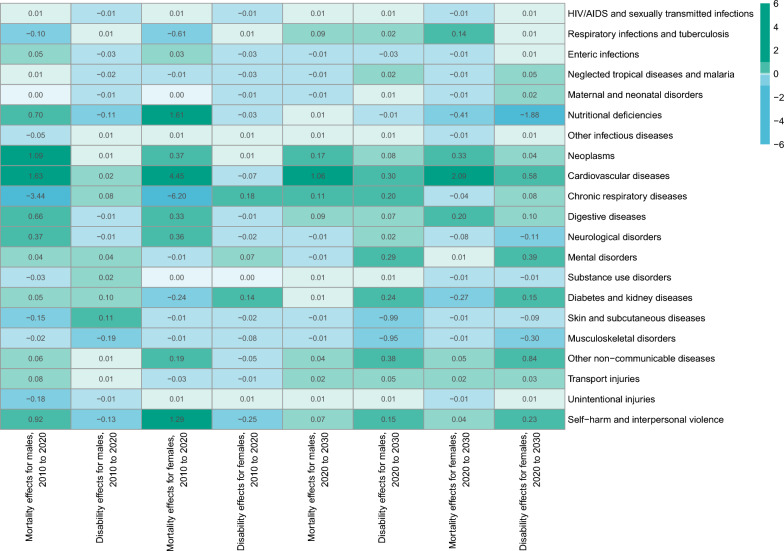
Decomposition of changes in health-adjusted life expectancy from 2010 to 2030, by cause and sex

Figure 4 shows the cause-specific effects on mortality and disability, some of which resulted in a loss of more than 0.1 years of HALE among the population aged over 60 years. Between 2010 and 2020, the mortality effects for males included chronic respiratory diseases (− 3.44 years), self-harm and interpersonal violence (− 0.18 years), skin and subcutaneous diseases (-0.15 years), and respiratory infections and tuberculosis (− 0.10 years). The disability effects for males included musculoskeletal disorders (− 0.19 years), unintentional injuries (− 0.13 years), and nutritional deficiencies (− 0.11 years). The mortality effects for females included chronic respiratory diseases (− 6.20 years), respiratory infections and tuberculosis (− 0.61 years), and diabetes and kidney diseases (− 0.24 years). The disability effects for females included unintentional injuries (− 0.25 years). From 2020 to 2030, the disability effects for males include skin and subcutaneous diseases (− 0.99 years), and musculoskeletal disorders (− 0.95 years). The mortality effects for females include nutritional deficiencies (− 0.41 years), and diabetes and kidney diseases (− 0.27 years). The disability effects for females include nutritional deficiencies (− 1.88 years), musculoskeletal disorders (− 0.30 years), and neurological disorders (− 0.11 years). The online Appendix Tables S5A and S5B provide detailed results on the decomposition of HALE, including the proportion of cause-specific effects in the total HALE change.

### Decomposition of changes in disability-adjusted life years from 2010 to 2030

**Fig. 5:**
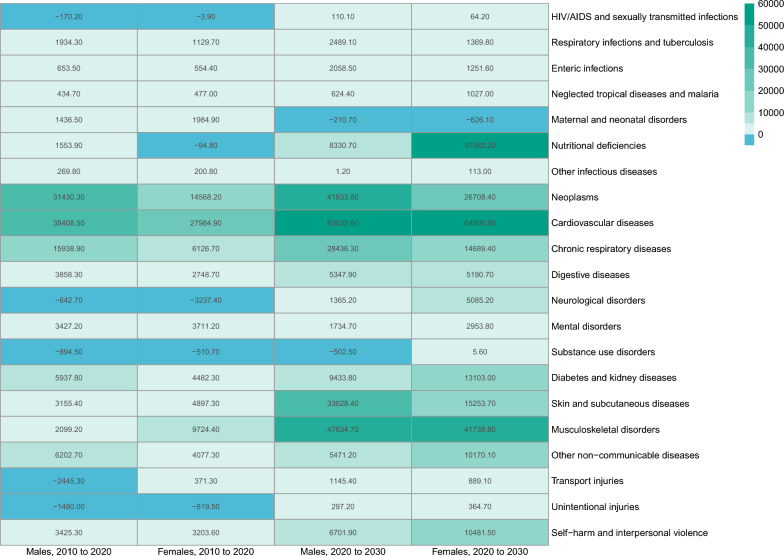
Disability-adjusted life years attributable to changes in age structure from 2010 to 2030, by cause and sex

Between 2010 and 2020, the increase in DALY attributable to changes in age structure accounted for 72.01% (114.5 thousand person-years) of the total increase in DALY for males. The top five causes were cardiovascular diseases (38.4 thousand person-years), neoplasms (31.4 thousand person-years), chronic respiratory diseases (15.9 thousand person-years), other non-communicable diseases (6.2 thousand person-years), and diabetes and kidney diseases (5.9 thousand person-years). The increase in DALY attributable to changes in age structure accounted for 46.68% (81.6 thousand person-years) of the total increase in DALY for females. The top five causes were cardiovascular diseases (28.0 thousand person-years), neoplasms (14.6 thousand person-years), musculoskeletal disorders (9.7 thousand person-years), chronic respiratory diseases (6.1 thousand person-years), and skin and subcutaneous diseases (4.9 thousand person-years). From 2020 to 2030, the increase in DALY attributable to changes in age structure accounted for 102.93% (186.3 thousand person-years) of the total increase in DALY for males. The top five causes are cardiovascular diseases (63.5 thousand person-years), neoplasms (41.8 thousand person-years), chronic respiratory diseases (28.4 thousand person-years), diabetes and kidney diseases (9.4 thousand person-years), and nutritional deficiencies (8.3 thousand person-years). The increase in DALY attributable to changes in age structure accounted for 100.99% (182.8 thousand person-years) of the total increase in DALY for females. The top five causes are cardiovascular diseases (64.8 thousand person-years), neoplasms (26.7 thousand person-years), skin and subcutaneous diseases (15.3 thousand person-years), chronic respiratory diseases (14.7 thousand person-years), and diabetes and kidney diseases (13.1 thousand person-years). **(**Fig. 5**)** The online Appendix Tables S6A, S6B, S6C, and S6D provide detailed results on the decomposition of DALY, including the change in DALY due to age structure, population size, and all other reasons with 95% *UIs*.

### Scenario analysis

The result of Scenario 1 shows that by 2030, the male YLL is projected to increase by 319.6 thousand person-years (45.0%) and YLD by 127.5 thousand person-years (31.8%) compared to 2020. The female YLL is expected to increase by 234.5 thousand person-years (54.7%) and YLD by 178.1 thousand person-years (33.8%) compared to 2020. In Scenario 2, the male YLL in 2030 is anticipated to decrease by 21.0 thousand person-years (− 2.9%), while YLD is projected to increase by 50.8 thousand person-years (12.7%) compared to 2020. The female YLL in 2030 is expected to decrease by 5.6 thousand person-years (− 1.3%), with an increase in YLD of 77.4 thousand person-years (14.7%) compared to 2020. (online Appendix Table S7 and online Appendix Figure S3) The online Appendix Tables S8A, S8B, S8C, and S8D provide the absolute number and percentage of increase in the disease burden in 2030 compared to 2020 by cause for Scenario 1 and Scenario 2 with 95% *UIs*.

**Fig. 6:**
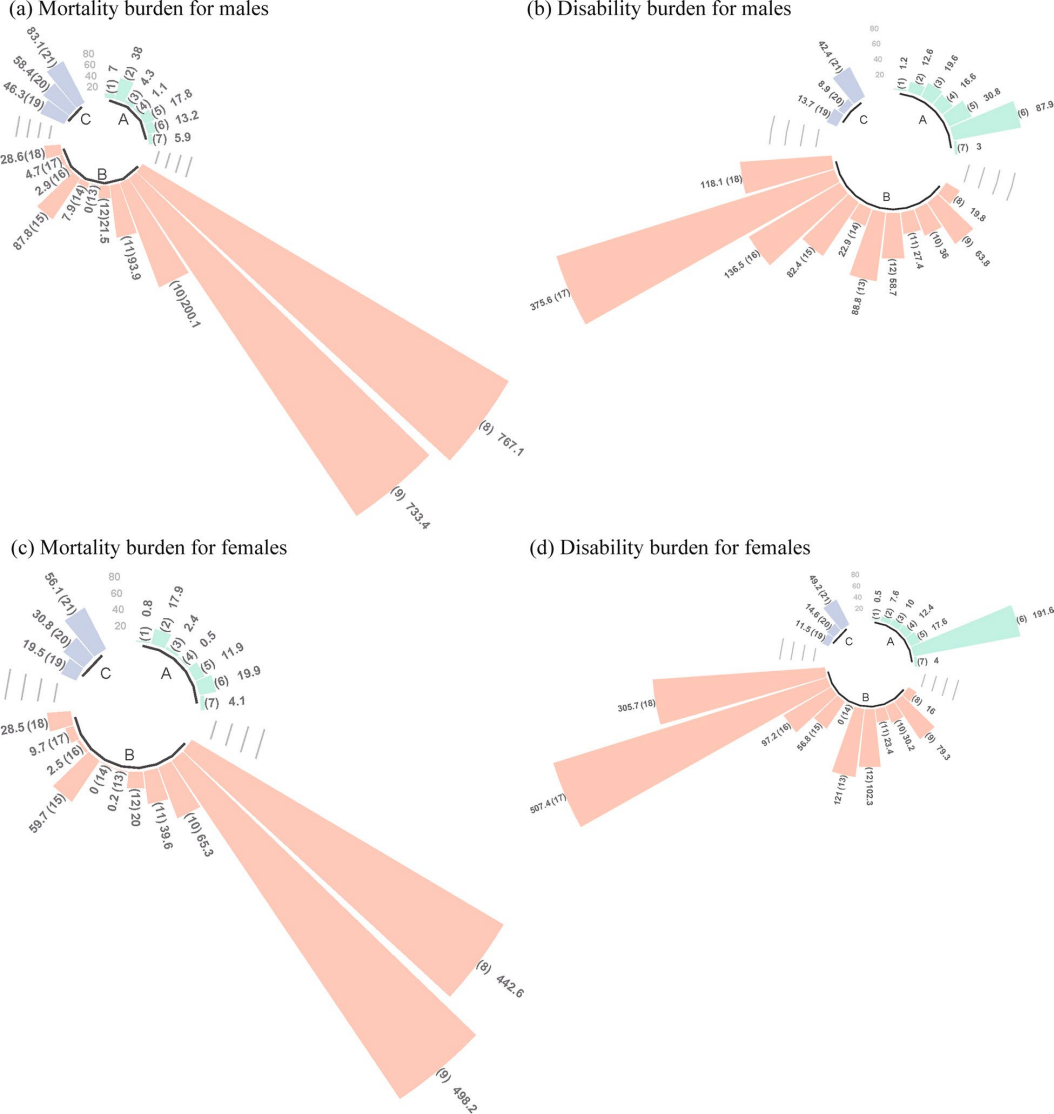
Expected reductions in the burden of disease in 2030 compared to 2020, by cause and sex.* Note*: The 3 categories of Level 2 causes include (A) to (C). The 21 categories of Level 3 causes include (1) to (21). The causes of the correspondence between letters and numbers are shown in the online Appendix Table S2.

Figure 6 shows the results of Scenario 3. The expected male YLL and YLD rates in 2030 are projected to be 12,306.3 and 6,930.7 person-years per 100,000 people, requiring reductions of 15.3% and 15.4% (2,223.2 and 1,266.7 person-years per 100,000 people), respectively, compared to 2020. The expected female YLL and YLD rates in 2030 are projected to be 7,310.1 and 8,989.3 person-years per 100,000 people, necessitating reductions of 15.4% and 15.6% (1,330.0 and 1,658.3 person-years per 100,000 people), respectively, compared to 2020. The online Appendix Tables S9A and S9B provide the expected burden of disease in 2030 and the reduction compared to 2020 with 95% *UIs*.

## Discussion

A proactive response to population aging is a key factor in determining a country's long-term economic growth momentum [23]. The health-related losses associated with an aging population not only place a heavy physical, mental, and economic burden on individuals, but also put a constant strain on healthcare systems. Promoting the development of a healthy aging system is, therefore, of great value [24]. Identifying the major health losses due to population aging is important for developing successful response strategies. Given this challenge, this study combined two health indicators, HALE and DALY, and proposed an attribution analysis framework to systematically investigate how population aging affects the quality of life and disease burden. We applied this framework to regional populations to comprehensively analyze the health impacts of population aging from two perspectives. To the best of our knowledge, this is the first study to quantitatively attribute and predict shifts in epidemic patterns of disease and injury due to population aging. Our proposed framework serves as an important top-level design to guide practice, allowing comparisons of the health effects of population aging across different time periods and regions.

### Impacts of population aging on quality of life

This study found that the decline in premature deaths in both sexes was the main determinant of HALE growth during the period 2010 to 2020, with people aged 60 years and over contributing more than half of this growth. This finding aligns with the national-level trends observed from 1990 to 2013, where improvements in HALE were similarly attributed to reductions in premature mortality among older adults [19]. With the transition in population health, the leading cause of the improvement in death levels has shifted from a significant increase in newborn survival to a significant increase in life expectancy among older adults, who now play a major role in extending both life expectancy and HALE [25]. However, this study also highlights that the positive effect of improved disability levels on the growth of HALE remains minimal, resulting in HALE failing to keep pace with the growth rate of life expectancy. This growing disparity indicates that longer life expectancy increasingly coincides with illness or disability, intensifying the demand for healthcare and long-term care among the elderly. This trend imposes substantial  economic costs and intangible burdens on individuals, families, and society [26]. In the long term, mortality reduction tends to decelerate as it approaches a lower threshold. For regions with lower baseline population health, higher mortality theoretically has more potential for improvement and may achieve a faster rate of decline. In more socially and economically developed regions, mortality may reach a  plateau that is challenging to lower further [27]. Comparing the results of this study with the gross mortality from all diseases and injuries in China reported by the GBD, the mortality in Guangzhou was significantly lower than the national level during the study period. However, the rate of decline was also slower than the national level, suggesting that the future causes of population health in advanced regions may gradually shift from death to disability.

The projection of future HALE indicates a steady increase in HALE for both sexes over the period 2020 to 2030. During this period, the reduction in premature deaths among people aged 85 years and over is expected to become the main contributor to the all-age mortality effect. However, the relative reduction in premature deaths among other age groups will limit the all-age mortality effect, preventing it from exceeding historical trends. This phenomenon may align with the relationship between life expectancy and mortality, which follows a logistic curve. As  life expectancy improves, the rate of increase gradually slows and eventually approaches zero, stablizing at a fixed constant. Based on this principle, some researchers have proposed that there is a natural physiological limit for human beings in the absence of external risk factors. However, the onset of chronic diseases can be delayed or even prevented by adopting health lifestyle choices such as reducing tobacco and alcohol consumption, maitaining a healthy weight, increasing physical activity and so on. These measures can compress the period between the onset of disease and death [28]. The findings of this study are also consistent with these theories. If the mortality effect for both sexes does not increase significantly, the proportion of the disability effect of all ages and the elderly population in the overall effect will increase. Notably, the contribution of disability improvement among elderly women to the growth of HALE shifts from negative to positive, suggesting that a future trend of morbidity compression in advanced regions, where the quality of life for older populations is likely to improve.

### Impacts of population aging on burden of disease

This study also found that the increase in DALY attributable to population size growth and age structure changes for both sexes between 2010 and 2020 outweighed the decrease from all other factors, resulting in a net increase in total DALY. Population size growth was the main driver of the increase in DALY, similar to the global trend in previous studies [29, 30]. Ongoing population change is one of the hallmarks of the fourth stage of epidemiology, in which chronic degenerative diseases are the main health threats. Improvement in disease prevention and control technology, as well as other external population factors, are delaying the onset of disease, reducing post-disease mortality and increasing the proportion of older people in the population age structure. The size of the population only increases over time, so the aging of the population is the main contributor to the accumulation of the disease burden [31]. Data from the National Population Census indicate that China began entering the fourth stage of health transformation during the period 2010 to 2020, which has been influenced by macro- and micro-level factors such as the continuous promotion of healthy aging policies, the gradual improvement in family and community environments suitable for aging populations, and the continuous improvement of people's health awareness and lifestyles [32]. This study also confirmed that under the guidance of the Chinese central government's top-level strategies to actively manage population aging, the impacts of population aging on the burden of disease in developed regions reflected the characteristics of the fourth stage of health transformation. Besides, DALY attributable to age structure accounted for a substantial proportion in both sexes, second only to the contribution of population growth.

The projection of future DALY suggests that the net increase in DALY for both sexes in the period 2020–2030 will exceed the historical trends, with total DALY continuing to accumulate over time. Unlike HALE, the accumulation of DALY is influenced by both population changes and the prevalence intensity of different causes, meaning there is no upper limit to DALY accumulation. Consequently, the healthcare system will face an increasing burden of disease. In addition, the DALY attributable to age structure changes will surpass not only the effects of population growth but also all other causes, suggesting that age structure change has transitioned from being a secondary to a primary driver of DALY accumulation.

### Translating evidence into health policy and practice

The attribution of population aging to HALE and DALY in this study can be interpreted through two complementary aspects of health measurement. Attributed HALE examines the per capita health loss in a hypothetical population. It is a health expectancy measure calculated by applying the real-world prevalence intensity of causes, expressed as a percentage of the disease spectrum, to a hypothetical cohort within the life table. The attributed HALE, therefore, highlights that improving the health of the older population can effectively extend the average healthy lifespan. The attributed DALY examines the cumulative loss of health in the real population, which accrues as the age structure shifts toward an older demographic. Attributive DALY, thus, represents the excess burden of disease that must be managed and controlled by the healthcare system during the period. The attributed HALE identifies the prevalence intensity of specific causes and their health effects from the perspective of the elderly population, while the attributed DALY identifies the impacts of changes in age structure of the population on the burden of disease from the perspective of societal development.

Empirical evidence indicates that the five leading causes of disease burden attributable to changes in age structure are all non-communicable diseases (NCDs). This trend is projected to persist in the future. The primary prevention of NCDs requires the improvement in population health literacy and the creation of a health-promoting environment [33, 34]. The efficacy of primary prevention is contingent upon the willingness of the population to engage in preventive measures [35]. For the elderly, offline intervention and health support tools are more suitable [36]. A health-promoting lifestyle can also improve the condition of elderly individuals with NCDs and delay the onset of complications [37]. The establishment of health records and the provision of medical follow-up services by community health centers represent effective health-promoting lifestyle interventions [38]. In addition, the burden of several interrelated diseases is projected to increase substantially, and the co-occurrence of these diseases (multimorbidity), such as nutritional deficiencies and musculoskeletal disorders, must be taken into account [39]. Aging and degeneration of the digestive system can lead to reduced nutrient intake or utilization, which in turn increases the risk of musculoskeletal disorders [40, 41]. Integrating targeted dietary advice and nutritional support into the musculoskeletal treatment program is an effective way to reduce the comorbidity of these two diseases [42]. Multimorbidity usually involves different combinations of risk factors or pathobiological mechanisms, which may alter the benefits and effectiveness of health interventions [43, 44]. An evidence-based, fundamental restructuring of interventions for older people with multimorbidity is essential to ensure their full integration into active aging practices [45].

### Limitations

A major limitation of this study is that it did not quantify the intensity and direction of the effects of population aging on the burden of multimorbidity. The probability of related diseases co-occurring is often much higher than the product of the probabilities of individual diseases, and the burden of multimorbidity may also be higher than the sum of the burdens of the individual diseases. However, this study included complete categories of causes and the findings hightlight key contributors to a substantial burden of disease, which may also overlap and interact. Given that the essence of the burden of disease is to obtain comparable relative importance of different causes using a consistent measurement framework, this study can still provide conservative estimates of the burden of different diseases and injuries, indicating the potential patterns of multimorbidity, and providing an important decision-making basis for formulating public policies to actively cope with population aging.

## Conclusions

This study has proposed an attribution framework for assessing the health effects of population aging from the two dimensions: quality of life and burden of disease. This framework includes a complete category of disease and injury and reveals the heterogeneity of the health effects of population aging across different causes and between sexes. Its theoretical basis can be used as a reference paradigm for quantifying the health effects of population aging in other regions and populations. A correct understanding of the significant changes in the health effect of population aging is essential to ensure that aging policies and pension systems are designed to meet the society's needs [46]. In the future, it will be necessary to carry out comprehensive attribution analyses of the health effects of population aging at both national and regional levels. Such anlyses should aim to identify major age-related diseases and injuries, and to respond to the challenges of population aging and seize the opportunities of population aging from the perspective of local conditions and precise policies.

## Supplementary Information

Additional file 1.

## Data Availability

The data that support the findings of this study are available from the Guangzhou Centre for Disease Control and Prevention. Restrictions apply to the availability of these data, which were used under license for this study. Data are available from the corresponding authors with the permission of the Guangzhou Centre for Disease Control and Prevention. The new datasets created or analyzed in this study that support the findings of this study are available in the supplementary material of this article.

## Abbreviations

HALE: Health-adjusted life expectancy
DALY: Disability-adjusted life years
YLL: Years of life lost
YLD: Years lived with disability
GBD: Global Burden of Disease Study
BAPC: Bayesian age-period-cohort model
UI: Uncertainty intervals
