# Supplementary material for: Effects of population aging on quality of life and disease burden: a population-based study
Source: Glob Health Res Policy. 2025 Jan 14;10:2. doi: 10.1186/s41256-024-00393-8 (PMC11731452; doi:10.1186/s41256-024-00393-8)
Supplement: Supplementary file 1 — Additional file 1. [file 41256_2024_393_MOESM1_ESM.pdf]

Effects of population aging on quality of life and disease burden: a population-based study

Appendix

Appendix Text S1. Method of redistributing garbage code 1

Appendix Text S2. Estimation method for Health-adjusted life expectancy and Disability-adjusted life years 2

Appendix Text S3. Bayesian age-period-cohort regression model 4

Appendix Text S4. Population prediction model based on the cohort-component method......6

Appendix Text S5. Decomposition method of Health-adjusted life expectancy......9

Appendix Text S6. Decomposition method of Disability-adjusted life years 11

Appendix Text S7. Mathematical expression of scenario analysis......13

Appendix Figure S1. Population aging process from 2010 to 2020 14

Appendix Figure S2. Decomposition of changes in Health-adjusted life expectancy from 2010 to 2030, by age group......15

Appendix Figure S3. The absolute number of changes in the disease burden in 2030 compared to 2020 16

Appendix Table S1. Summary of the methodological details in this study 17

Appendix Table S2. Cause and sequelae hierarchy......18

Appendix Table S3. Mapping of ICD-10 to cause and sequelae 19

Appendix Table S4. Mapping of ICD-10 to garbage code......22

Appendix Table S5A. Decomposition of changes in Health-adjusted life expectancy from 2010 to 2020, by cause and sex 23

Appendix Table S5B. Decomposition of changes in Health-adjusted life expectancy from 2020 to 2030, by cause and sex 24

Appendix Table S6A. Decomposition of changes in Disability-adjusted life years for males from 2010 to 2020 (95% *UIs*) 25

Appendix Table S6B. Decomposition of changes in Disability-adjusted life years for females from 2010 to 2020 (95% *UIs*) 26

Appendix Table S6C. Decomposition of changes in Disability-adjusted life years for males from 2020 to 2030 (95% *UIs*) 27

Appendix Table S6D. Decomposition of changes in Disability-adjusted life years for females from 2020 to 2030 (95% *UIs*) 28

Appendix Table S7. The absolute number and percentage of changes in the disease burden in 2030 compared to 2020 (95% *UIs*) 29

Appendix Table S8A. In Scenario 1, the absolute number and percentage of increase in the disease burden for males in 2030 compared to 2020, by cause (95% *UIs*) 30

Appendix Table S8B. In Scenario 1, the absolute number and percentage of increase in the disease burden for females in 2030 compared to 2020, by cause (95% *UIs*) 32

Appendix Table S8C. In Scenario 2, the absolute number and percentage of increase in the disease burden for males in 2030 compared to 2020, by cause (95% *UIs*) 34

Appendix Table S8D. In Scenario 2, the absolute number and percentage of increase in the disease burden for females in 2030 compared to 2020, by cause (95% *UIs*) 36

Appendix Table S9A. Expected burden of disease for males in 2030 and the reduction compared to 2020, by cause (95% *UIs*) 38

Appendix Table S9B. Expected burden of disease for females in 2030 and the reduction compared to 2020, by cause (95% *UIs*) 40

Appendix Text S1. Method of redistributing garbage code

For ICD-10 codes representing unclear symptoms or signs in the underlying cause of death, we redistributed them according to the GBD garbage code list and previous research methods as follows.

$$n_{ij}^{s,after} = \left(n_{ij}^{s,before} - n_{ij}^{garbage}\right) \times \left(1 + n_{ij}^{garbage} / N_{ij}^{t,before}\right)$$

Where i,j represent sex and age group respectively, $n^{s,before}$ and $n^{s,after}$ represent the total number of a specific cause before and after redistribution, $N^{t,before}$ represents the total number of all-cause before redistribution, and $n^{garbage}$ represents the total number of garbage codes for a specific cause.

Appendix Text S2. Estimation method for Health-adjusted life expectancy and Disability-adjusted life years

We used the number of deaths in Guangzhou and the reference life table from the Global Burden of Disease Study 2019 (GBD 2019) to calculate the years of life lost (YLL), and the method proposed by the WHO to calculate the years lived with disability (YLD). First, YLL was calculated based on the all-cause death dataset in Guangzhou.

$$YLL_{ij} = \sum_{1}^{w} N_{ij} \times L_{ij}$$

Where i, j represent sex and age group respectively, N is the number of people in a certain age group and gender group who die from a certain cause, and L is the value of life lost in a certain age group (the life expectancy value corresponding to this age group in the life table). This study used the reference life table obtained from the GBD 2019, which was based on the lowest mortality observed in all age groups globally, to construct the standard life expectancy from the highest value of life expectancy in each age group globally.

Second, we calculate YLD. It is extremely difficult to calculate accurate YLD because of the lack of basic data necessary to calculate them. The WHO has studied the internal relationship between YLD and YLL in various regions of the world and an indirect method for calculating YLD was developed.

$$YLD_{study\ area\ ij} = \frac{YLD_{reference\ area\ ij}}{YLL_{reference\ area\ ij}} \times YLL_{study\ area\ ij}$$

The reference area for this study is China. The YLD-YLL ratios of China from 2010 to 2019 were obtained from the public database of GBD, and the 2020 estimate uses the 2019 reference value.

Third, we used Sullivan’s method to incorporate YLD into life tables, Health-adjusted life expectancy (HALE) is calculated by taking into account the loss of life due to disease or disability in each age group.

$$HALE_{ij} = \frac{1}{l_{ij}} \sum_{j}^{w} L_{ij} \times (1 - YLD_{ij})$$

Where, l_{ij} represents the number alive at the start of the interval for sex i and age j in the abbreviated life table, L_{ij} represents the number of years lived in the interval, and w represents the last age group.

Finally, Disability-adjusted life years (DALY) are defined as the sum of YLL and YLD.

$$DALY = YLL + YLD$$

Appendix Text S3. Bayesian age-period-cohort regression model

The age-specific epidemiological indicators (mortality, YLL rates, and YLD rates) were projected using the Bayesian age-period-cohort (BAPC) regression model with the Integrated Nested Laplace Approximations (INLA) method. The Bayesian approach attributes separate effects to age, period, and cohort, and extrapolates these effects to make projections. Due to the expectation that effects adjacent in time might be similar, smoothing priors are commonly used for age, period, and cohort effects and to project posterior age-specific epidemiological indicators. A standard choice is the second-order random walk (RW2). According to this model, each point of effect is predicted by linear extrapolation from its two immediate predecessors, plus a random variance from a normal distribution with mean zero.

In the absence of potential overdispersion, the observed epidemiological indicators y_{ij} in age group i at time point j can be assumed to be Poisson distributed with mean $n_{ij}\lambda_{ij}$, where n_{ij} denotes the corresponding person-time of exposure, assumed to be known. In this study, the age index i from 1 to $i = 19$ while the period index j from 1 to $j = 10$. The linear predictor $\eta_{ij} = log(\lambda_{ij})$ is commonly specified as $log(\lambda_{ij}) = \mu + \alpha_i + \beta_j + \gamma_k$. Here, μ represents the general level (intercept), and α_i, β_i, γ_k denote age, period, and cohort effects, respectively. The cohort index k depends on the age group and period index, but also on the width of the age group and period intervals. Here, it is defined as $k = M \times (I - i) + j$, where M indicates that the age group intervals are M times wider than the period interval. Consider the age effects, say, then the RW2 prior is given by

$$f(\alpha|\kappa_\alpha) \propto \kappa_\alpha^{\frac{I-2}{2}} exp\left(-\frac{\kappa_\alpha}{2}\sum_{i=3}^{I}(\alpha_i - 2\alpha_{i-1} + \alpha_{i-2})^2\right) = \kappa_\alpha^{\frac{I-2}{2}} exp\left(-\frac{1}{2}\alpha^T Q\alpha\right)$$

$$Q = \mathcal{K}_\alpha \begin{pmatrix} 1 & -2 & 1 & & & & \\ -2 & 5 & -4 & 1 & & & \\ 1 & -4 & 6 & -4 & 1 & & \\ & \ddots & \ddots & \ddots & \ddots & \ddots & \\ & & 1 & -4 & 6 & -4 & 1 \\ & & & 1 & -4 & 5 & -2 \\ & & & & 1 & -2 & 1 \end{pmatrix}$$

where κ_α^{-1} denotes the variance parameter. Note that Q is not of full rank so the RW2 is an example of an intrinsic Gaussian Markov field. We are interested in the mortality or incidence rates for the same age groups but t periods ahead into the future, which means

$$log(\lambda_{i,J+t}) = \mu + \alpha_i + \beta_{J+t} + \gamma_{k+t} + z_{i,J+t}$$

For this purpose, we need to extrapolate the period and cohort effects following the structure of the RW2 model. To be more specific, assume we have data up to period $J \geq 2$, then the period effect at period $J + 1$ will have the conditional distribution

$$\beta_{J+1} | \beta_1, \cdots, \beta_J, \kappa_\beta \sim \mathcal{N}(2\beta_J - \beta_{J-1}, \kappa_\beta^{-1})$$

If $t > 1$, that is, interest lies not in one-step ahead but t-steps ahead forecasts, this leads to

$$\beta_{J+t} | \beta_1, \cdots, \beta_J, \kappa_\beta \sim \mathcal{N}[(1+t)\beta_h - t\beta_{h-1}, \kappa_\beta^{-1}(1 + 2^2 + \cdots + t^2)]$$

Thus, the conditional mean is given by a linear extrapolation of the last two period effects with cubically increasing variance. Analogously, projections for the cohort effects are obtained.

To construct the BAPC model, we introduced the Lexis diagram to the organization of our data. The Lexis diagram consists of population estimates and epidemiological indicators by year and age. Data are given by single calendar year and 19 age groups (>1, 1 - 4, 5 - 9,⋯, 80 - 84, 85+ years). Within the text files, the rows represent periods in increasing order and the columns age groups from young to old. That said, each row represents one period, and each column (besides the first, which shows the year label) represents one age group.

Appendix Text S4. Population prediction model based on the cohort-component method

This study takes into account the policy direction of China, the current development situation of Guangzhou, and the theories of previous studies to set the parameters of population change, including three prediction scenarios of high, medium, and low population development. The population projection assumptions in this study include (a) the birth and death patterns of the population will remain relatively stable over the projection period, (b) the steady and orderly development of the cities over the projection period will not cause major changes in population flow patterns in the short term. Specifically, the parameters of prediction scenarios are set as follows.

(1) Initial population: The population distribution in 2020 was used as the initial population for the three prediction scenarios.

(2) Mortality level: The low, medium, and high prediction scenarios were extrapolated to 2030 using the lower, middle, and upper for 95% confidence intervals of the average annual percentage change in life expectancy from 2010 to 2020, respectively.

(3) Mortality pattern: All three prediction scenarios used the United Nations life table.

(4) Fertility level: The total fertility rate is the average number of children that each woman can have in a population of women of childbearing age between 15 and 49 years and is calculated by summing the age-specific fertility rates of the population. Since the early 1990s, the number of births registered in China's National Population Census and National Population Sample Survey, as well as the total fertility rate calculated from the number of births, have been significantly lower, which may be due to underreporting of birth registration. Although the true total fertility rate is unclear, relatively accurate crude birth rates can be obtained. According to the definition of crude birth rate and total fertility rate, the two are usually linked by a specific parameter. First, this parameter is estimated using specific data and then, according to the derived functional relationship, the total fertility rate is estimated using the known crude birth rate.

$$K = \sum_{x=15}^{49} (h_x \times C_x)$$

$$\beta = CBR/K$$

Where, h_x is the standardized fertility rate at age x (fertility rate of women at age x / total fertility rate), C_x is the proportion of women at age x in the total population (number of women at age x / total population), CBR is the crude birth rate, and β is the total fertility rate.

After obtaining the true historical total fertility rate, the Bayesian hierarchical model for predicting the total fertility rate developed by the United Nations Population Division was used to estimate the total fertility rate up to 2030. The United Nations Population Division's projections of fertility levels are primarily based on the fertility transition theory. According to the fertility transition theory, the fertility transition can be divided into three stages. The first stage is the initial phase of the fertility transition, which is characterized by a stable and high fertility rate, i.e. the total fertility rate fluctuates above 6. The second stage is the fertility transition period when a country's total fertility rate gradually declines from around 5.5 to the replacement level of 2.1 or below. The third stage is the post-fertility transition period when the total fertility rate rises from below 2.1 to around 2.1. The Bayesian hierarchical model for predicting total fertility rate consists of several levels, the first level contains fertility data for the region that is predicted, and the second level uses average data for countries around the world, assuming that the unknown parameters in each region are a sample of the probability distribution composed of the past fertility transition histories of all countries in the world. In this study, the lower, middle, and upper limits of the 95% confidence intervals of the model output were used for the low, medium, and high prediction scenarios, respectively.

(5) Fertility pattern: The age-specific fertility distribution from the 1% National Population Sample Survey in 2015 was used for the low, medium, and high prediction scenarios.

(6) Newborn sex ratio: The sex ratio at birth is the number of male newborns for every 100 live female newborns. The National Plan on Population Development

(2016-2030), issued by China's State Council in January 2017, emphasizes the need to "strengthen the management of the sex ratio at birth and promote gender equality in society" and sets the expected development target for the population structure as "the sex ratio at birth should not exceed 112 by 2020 and should be reduced to 107 by 2030". The sex ratio at birth in Guangzhou in 2020 is 112.97, which is slightly higher than the national target for 2020, but the gap with the 2030 plan target is not very large. Therefore, it is assumed that the future development direction of Guangzhou's population structure can develop towards the expected target, and the sex ratio at birth is set at 112.97 in the starting year and 107 in 2030 for the three prediction scenarios. The linear interpolation method is used for the remaining years.

(7) Migration level: According to the Guangzhou Statistical Yearbook, the net migration rates of the population in Guangzhou from 2010 to 2020 are as follows: 10.26‰, 6.68‰, 5.58‰, 3.36‰, 5.21‰, 5.44‰, 5.12‰, 8.41‰, 15.33‰‰, 19.64‰, 17.81‰, and the average annual mobility is 9.35‰. The low, medium, and high prediction scenarios assume annual migration levels of 5‰, 10‰, and 15‰ respectively by 2030.

(8) Migration pattern: The age-specific migration distribution of the 1% National Population Sample Survey in 2015 in Guangzhou was used for the three prediction scenarios.

Appendix Text S5. Decomposition method of Health-adjusted life expectancy

First, HALE changes are decomposed into age-specific mortality and disability effects. The mortality effect refers to the change in HALE when mortality changes but disability remains unchanged. The disability effect refers to the change in HALE when only disability changes but death remains the same.

$$\triangle_n HALE_x = \frac{{}_nh_x^{t+i} \times {}_nL_x^{t+i} - {}_nh_x^t \times {}_nL_x^t}{l_a^t}$$

$${}_nh_x^t = 1 - {}_nd_x^t$$

$${}_nh_x^{t+i} = 1 - {}_nd_x^{t+i}$$

Where, $\triangle_n HALE_x$ represents the HALE change of age group $(x, x+n)$ at t and $t+i$ time points. ${}_nh_x^t$ and ${}_nh_x^{t+i}$ represent the proportion of the age group $(x, x+n)$ without disability at t and $t+i$ time points. ${}_nd_x^t$ and ${}_nd_x^{t+i}$ represent the total YLD per capita of the age group $(x, x+n)$ at t and $t+i$ time points. ${}_nL_x^t$ and ${}_nL_x^{t+i}$ represent the number of years lived in the age group $(x, x+n)$ at t and $t+i$ time points. l_a^t represents the number alive at the start of the 0 age group at t time points.

$$\triangle_n HALE_x = {}_nMOR_x + {}_nDIS_x$$

$${}_nMOR_x = \left(\frac{{}_nh_x^t + {}_nh_x^{t+i}}{2l_a^t}\right) \times \triangle_n L_x$$

$${}_nDIS_x = \left(\frac{{}_nL_x^t + {}_nL_x^{t+i}}{2l_a^t}\right) \times \triangle_n h_x$$

$$\triangle_n L_x = {}_nL_x^{t+i} - {}_nL_x^t$$

$$\triangle_n h_x = {}_nh_x^{t+i} - {}_nh_x^t$$

Where, ${}_nMOR_x$ represents the mortality effect in the age group $(x, x+n)$, and ${}_nDIS_x$ represents the disability effect in the age group $(x, x+n)$.

Secondly, the mortality and disability effects are further decomposed into the contribution of different causes.

$${}_nMOR_{xk} = {}_nMOR_x \times \frac{{}_nm_x^{t+i} \times {}_nf_{xk}^{t+i} - {}_nm_x^t \times {}_nf_{xk}^t}{{}_nm_x^{t+i} - {}_nm_x^t}$$

$$ {}_{n}DIS_{xk} = \left(\frac{ {}_{n}L_{x}^{t} + {}_{n}L_{x}^{t+i} }{ 2l_{a}^{t} } \right) \times \triangle_{n} h_{xk} $$

$$ \triangle_{n} h_{xk} = {}_{n}h_{xk}^{t+i} - {}_{n}h_{xk}^{t} $$

$$ {}_{n}h_{xk}^{t} = 1 - {}_{n}d_{xk}^{t} = 1 - {}_{n}d_{x}^{t} \times {}_{n}g_{xk}^{t} $$

$$ {}_{n}h_{xk}^{t+i} = 1 - {}_{n}d_{xk}^{t+i} = 1 - {}_{n}d_{x}^{t+i} \times {}_{n}g_{xk}^{t+i} $$

$$ \triangle_{n} h_{xk} = {}_{n}d_{x}^{t} \times {}_{n}g_{xk}^{t} - {}_{n}d_{x}^{t+i} \times {}_{n}g_{xk}^{t+i} $$

Where, ${}_{n}m_{x}^{t}$ and ${}_{n}m_{x}^{t+i}$ represent the total mortality of the age group $(x, x+n)$ at t and $t+i$ time points. ${}_{n}f_{xk}^{t}$ and ${}_{n}f_{xk}^{t+i}$ represent the proportion of mortality of specific cause k in the total mortality of the age group $(x, x+n)$ at t and $t+i$ time points. ${}_{n}g_{xk}^{t}$ and ${}_{n}g_{xk}^{t+i}$ the proportion of the YLD of specific cause k in the total YLD of the age group $(x, x+n)$ at t and $t+i$ time points.

Appendix Text S6. Decomposition method of Disability-adjusted life years

First, we then sorted and organized the data into the following format.

Age group	Year 1（$j = 1$）				Year 2（$j = 2$）			
	d_{i1}	n_{i1}	r_{i1}	s_{i1}	d_{i2}	n_{i2}	r_{i2}	s_{i2}
1	d_{11}	n_{11}	r_{11}	s_{11}	d_{12}	n_{12}	r_{12}	s_{12}
2	d_{21}	n_{21}	r_{21}	s_{21}	d_{22}	n_{22}	r_{22}	s_{22}
⋮	⋮	⋮	⋮	⋮	⋮	⋮	⋮	⋮
p	d_{p1}	n_{p1}	r_{p1}	s_{p1}	d_{p2}	n_{p2}	r_{p2}	s_{p2}
Total	D_1	N_1	R_1	$S_1 = 1$	D_2	N_2	R_2	$S_2 = 1$

Where, d_{ij} , n_{ij} , r_{ij} , and s_{ij} represent the age-specific number of DALY, age-specific population size, age-specific DALY crude rate, and proportion of age-specific population among the total population in the i age group and the j year respectively. D_{ij} , N_{ij}, R_{ij}, and S_{ij} represent the totals of the columns.

Then, the main effect of the three factors is represented by M_p, M_s, and M_m respectively, the two-way interaction of the three factors is represented by I_{ps}, I_{pm}, and I_{sm} respectively, and the three-way interaction of the three factors is represented by I_{pSm}, which can be calculated according to the following formula.

$$M_p = \sum_{i=1}^{p} (N_2 - N_1) \times s_{i1} \times r_{i1}$$

$$M_s = \sum_{i=1}^{p} N_1 \times (s_{i2} - s_{i1}) \times r_{i1}$$

$$M_m = \sum_{i=1}^{p} N_1 \times s_{i1} \times (r_{i2} - r_{i1})$$

$$I_{PS} = \sum_{i=1}^{p} (N_2 - N_1) \times (s_{i2} - s_{i1}) \times r_{i1}$$

$$I_{pm} = \sum_{i=1}^{p} (N_2 - N_1) \times s_{i1} \times (r_{i2} - r_{i1})$$

$$I_{sm} = \sum_{i=1}^{p} N_1 \times (s_{i2} - s_{i1}) \times (r_{i2} - r_{i1})$$

$$I_{psm} = \sum_{i=1}^{p} (N_2 - N_1) \times (s_{i2} - s_{i1}) \times (r_{i2} - r_{i1})$$

In the formula, subscripts p, s, and m indicate population size, age structure, and all other reasons respectively.

Finally, the contribution of each factor is represented by AS_{III}, PS_{III}, and ASR_{III} respectively, and is calculated according to the following formula.

$$AS_{III} = M_s + \frac{1}{2} I_{ps} + \frac{1}{2} I_{sm} + \frac{1}{3} I_{pSm}$$

$$PS_{III} = M_p + \frac{1}{2}I_{ps} + \frac{1}{2}I_{pm} + \frac{1}{3}I_{pSm}$$

$$ASR_{III} = M_m + \frac{1}{2}I_{pm} + \frac{1}{2}I_{sm} + \frac{1}{3}I_{pSm}$$

Appendix Text S7. Mathematical expression of scenario analysis

Three scenarios were developed to analyze the relative relationship between changes in the burden of premature death (YLL rates) and disability (YLD rates) and population change.

1. Scenario 1 explores the influence of population change on the burden of disease by assuming that YLL and YLD rates in 2030 remain at 2020 levels.

$$\mathrm{R} = \text{Rates of } \mathrm{DoB}_{2020} \times (\mathrm{POP}_{2030} - \mathrm{POP}_{2020})$$

2. Scenario 2 explores the influence of population change on the burden of disease by assuming that YLL and YLD rates in 2030 change from historical trends (modeling projections).

$$\mathrm{R} = \text{Rates of } \mathrm{DoB}_{2030} \times (\mathrm{POP}_{2030} - \mathrm{POP}_{2020})$$

3. Scenario 3 assumes that the health effects of population changes are offset in 2030 without increasing the absolute numbers of YLL and YLD, i.e. that the absolute numbers of YLL and YLD in 2030 remain at the 2020 level and that the expected levels of burden of premature death and disability are achieved relative to the levels to be controlled in 2020.

$$\mathrm{R} = \frac{\text{Absolute numbers of } \mathrm{DoB}_{2020} \times (\mathrm{POP}_{2030} - \mathrm{POP}_{2020})}{\mathrm{POP}_{2030} \times \mathrm{POP}_{2020}}$$

where the subscripts 2020 and 2030 represent the years, DoB represents YLL or YLD, and POP represents population distribution.

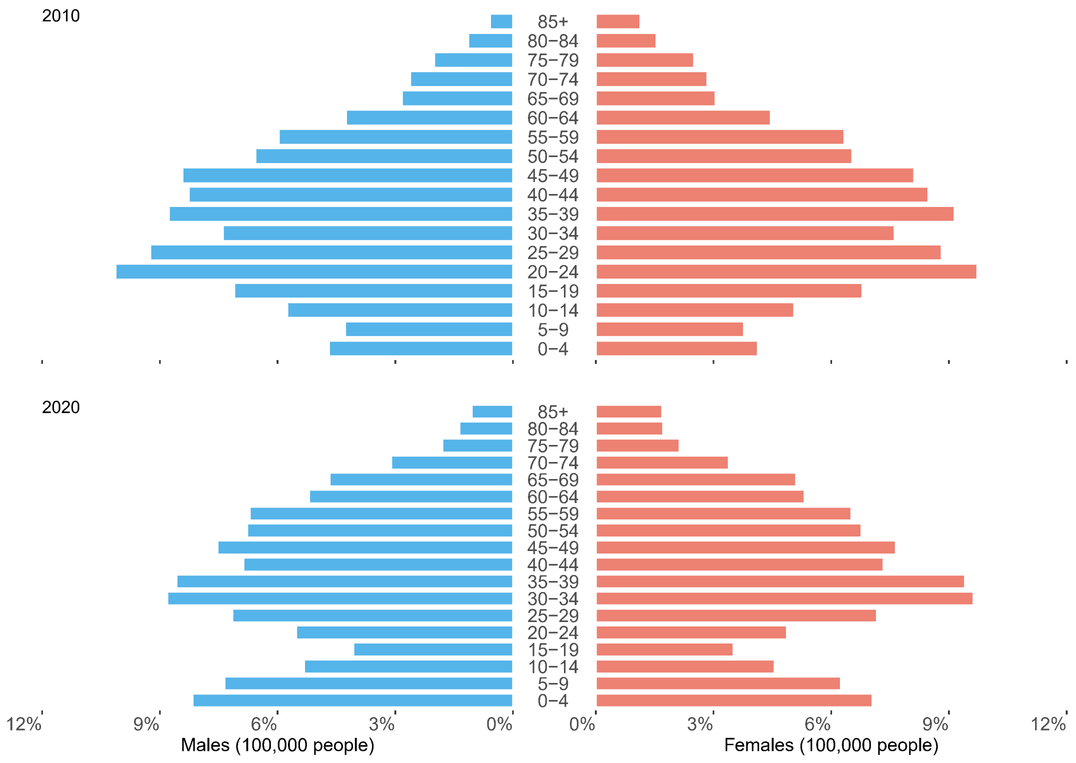

Appendix Figure S1. Population aging process from 2010 to 2020

A) 2010 — 2020

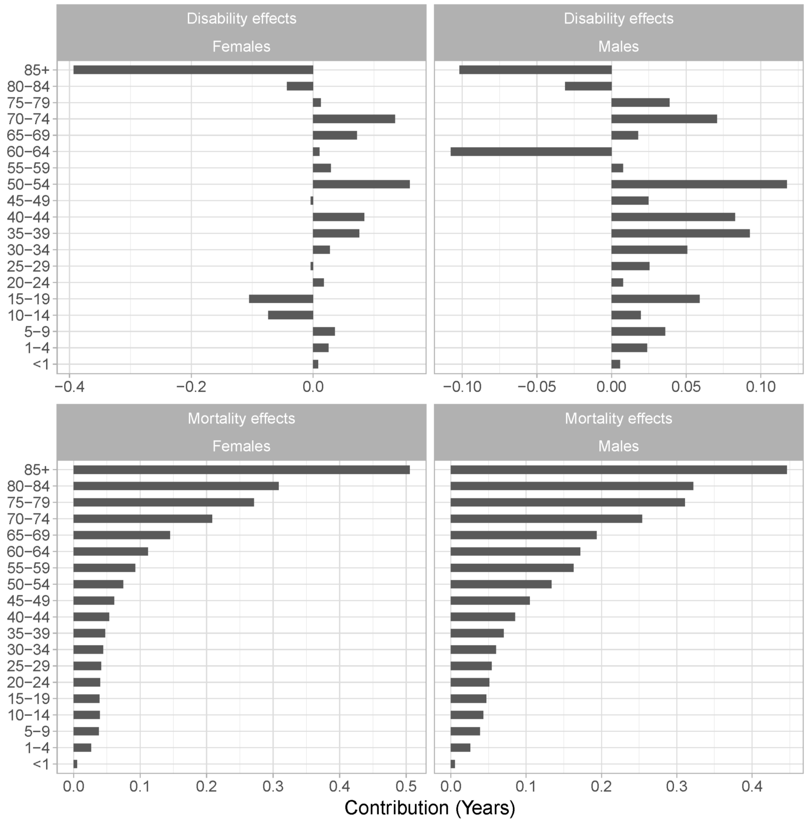

B) 2020 — 2030

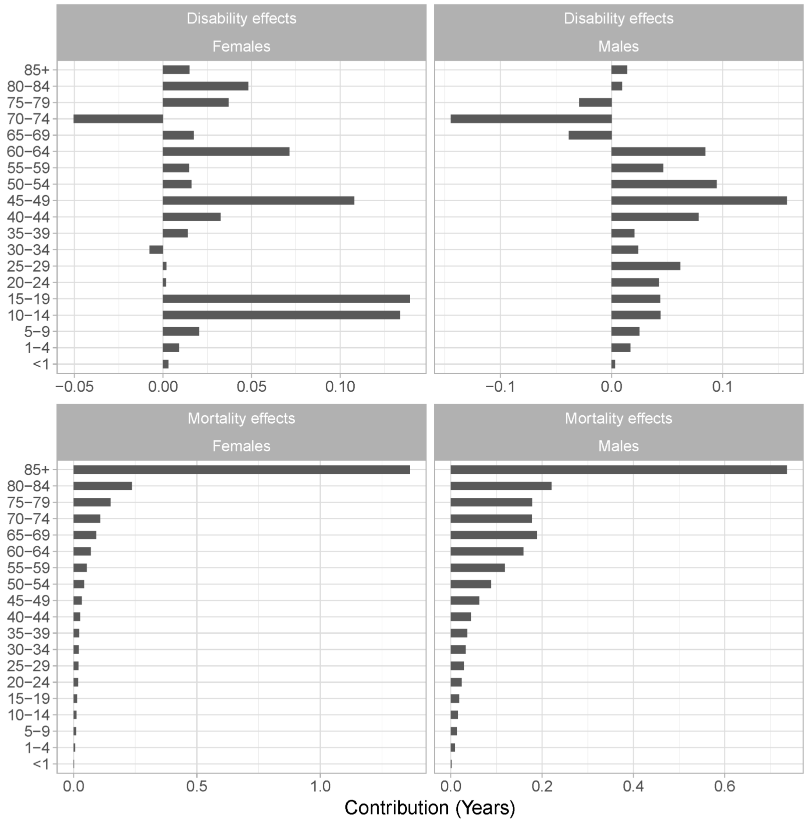

Appendix Figure S2. Decomposition of changes in Health-adjusted life expectancy from 2010 to 2030, by age group

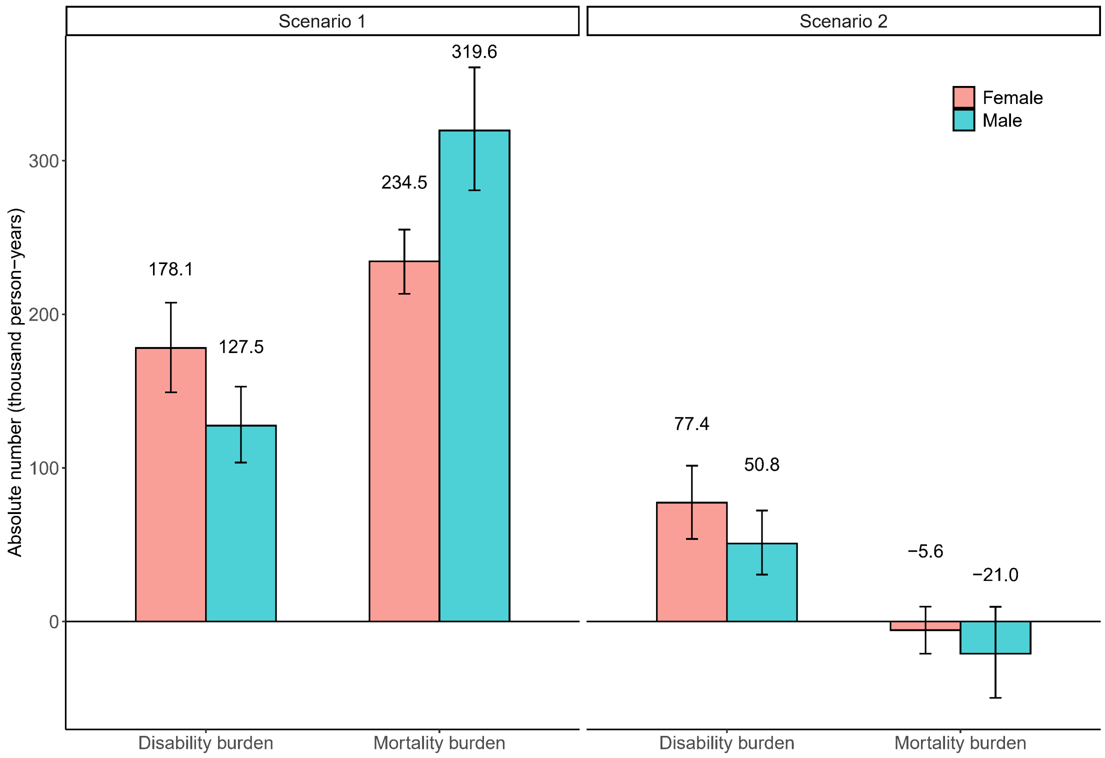

Appendix Figure S3. The absolute number of changes in the disease burden in 2030 compared to 2020

Appendix Table S1. Summary of the methodological details in this study

Methods	Tool/software used	Objects of the analysis	Note
Redistributing garbage code	Programming in R (v4.1.0)	Surveillance dataset on cause-of-death	Appendix Text S1
Measuring Health-adjusted life expectancy and Disability-adjusted life years	Programming in R (v4.1.0)	Surveillance dataset on cause-of-death and registered population dataset	Appendix Text S2
Bayesian age-period-cohort model	BAPC package (v0.0.36) based on R (v4.1.0)	Distribution of mortality and disability over the current period	Appendix Text S3
Population prediction model	PADIS-INT (v1.0); bayesTFR package (v7.3.0) based on R (v4.1.0)	Distribution of population change over the current period	Appendix Text S4
Decomposition model	Programming in R (v4.1.0)	Health-adjusted life expectancy and Disability-adjusted life years for the current period and the projection period	Appendix Text S5 and S6
Scenario analysis	Programming in R (v4.1.0)	Assumed future levels of disease burden and projected population distributions	Appendix Text S7

Appendix Table S2. Cause and sequelae hierarchy

Level 1	Level 2
**(A)**	**Communicable, maternal, neonatal, and nutritional diseases**
(1)	HIV/AIDS and sexually transmitted infections
(2)	Respiratory infections and tuberculosis
(3)	Enteric infections
(4)	Neglected tropical diseases and malaria
(5)	Maternal and neonatal disorders
(6)	Nutritional deficiencies
(7)	Other infectious diseases
**(B)**	**Non-communicable diseases**
(8)	Neoplasms
(9)	Cardiovascular diseases
(10)	Chronic respiratory diseases
(11)	Digestive diseases
(12)	Neurological disorders
(13)	Mental disorders
(14)	Substance use disorders
(15)	Diabetes and kidney diseases
(16)	Skin and subcutaneous diseases
(17)	Musculoskeletal disorders
(18)	Other non-communicable diseases
**(C)**	**Injuries**
(19)	Transport injuries
(20)	Unintentional injuries
(21)	Self-harm and interpersonal violence

Appendix Table S3. Mapping of ICD-10 to cause and sequelae

Level 2	ICD-10
HIV/AIDS and sexually transmitted infections	A50-A58, A60-A60.9, A63-A63.8, B20-B24.9, B63, F02.4, I98.0, K67.0-K67.2, M03.1, M73.0-M73.1
Respiratory infections and tuberculosis	A10-A14, A15-A19.9, A48.1, A70, B90-B90.9, B97.4-B97.6, H70-H70.9, J00-J02.8, J03-J03.8, J04-J04.2, J05-J05.1, J06.0-J06.8, J09-J15.8, J16-J16.9, J20-J21.9, J36-J36.0, J91.0, K67.3, K93.0, M49.0, N74.1, P23.0-P23.4, P37.0, U04-U04.9, U84.3
Enteric infections	A00-A00.9, A01.0-A09.9, A80-A80.9, K52.1, R19.7
Neglected tropical diseases and malaria	A68-A68.9, A69.2-A69.9, A75-A75.9, A77-A79.9, A82-A82.9, A90-A96.9, A98-A98.8, B33.0-B33.1, B50-B54.0, B55.0, B56-B57.5, B60-B60.8, B65-B67.9, B69-B72.0, B74.3-B75, B77-B77.9, B83-B83.8, K93.1, P37.1, U06-U06.9
Other infectious diseases	A20-A28.9, A32-A39.9, A48.2, A48.4-A48.5, A65-A65.0, A69-A69.1, A74, A74.8-A74.9, A81-A81.9, A83-A89.9, B00-B06.9, B10-B10.8, B15-B16.2, B17.0, B17.2, B19.1, B25-B27.9, B29.4, B33, B33.3-B33.8, B47-B48.8, B91, B94.1, B95-B95.5, D70.3, D89.3, F02.1, F07.1, G00.0-G00.8, G03-G03.8, G04-G05.8, G14-G14.6, G21.3, I00, I02, I02.9, I98.1, K67.8, K75.3, K76.3, K77.0, M49.1, M89.6, P35-P35.9, P37, P37.2, P37.5-P37.9, U82-U84, U85-U89, Z16-Z16.3
Maternal and neonatal disorders	C58-C58.0, N96, N98-N98.9, O00-O07.9, O09-O16.9, O20-O26.9, O28-O36.9, O40-O48.1, O60-O77.9, O80-O92.7, O96-O98.6, O98.8-P04.2, P04.5-P05.9, P07-P15.9, P19-P22.9, P24-P29.9, P36-P36.9, P38-P39.9, P50-P61.9, P70-P70.1, P70.3-P72.9, P74-P78.9, P80-P81.9, P83-P84, P90-P94.9, P96, P96.3-P96.4, P96.8
Nutritional deficiencies	D50.1-D50.8, D51-D52.0, D52.8-D53.9, E00-E02, E40-E46.9, E51-E61.9, E63-E64.0, E64.2-E64.9, M12.1
Neoplasms	C00-C13.9, C15-C22.8, C23-C25.9, C30-C34.9, C37-C38.8, C40-C41.9, C43-C45.9, C47-C54.9, C56-C57.8, C60-C63.8, C64-C67.9, C68.0-C68.8, C69.0-C69.8, C70-C73.9, C75-C75.8, C81-C86.6, C88-C91.0, C91.2-C91.3, C91.6, C92-C92.6, C93-C93.1, C93.3, C93.8, C94-C96.9, D00.1-D00.2, D01.0-D01.3, D02.0-D02.3, D03-D06.9, D07.0-D07.2, D07.4-D07.5, D09.0, D09.2-D09.3, D09.8, D10.0-D10.7, D11-D12.9, D13.0-D13.7, D14.0-D14.3, D15-D16.9, D22-D24.9, D26.0-D27.9, D28.0-D28.1, D28.7, D29.0-D29.8, D30.0-D30.8, D31-D36, D36.1-D36.7, D37.1-D37.5,

Level 2	ICD-10
	D38.0-D38.5, D39.1-D39.2, D39.8, D40.0-D40.8, D41.0-D41.8, D42-D43.9, D44.0-D44.8, D45-D47.9, D48.0-D48.6, D49.2-D49.4, D49.6, K62.0-K62.1, K63.5, N60-N60.9, N84.0-N84.1, N87-N87.9
Cardiovascular diseases	B33.2, G45-G46.8, I01-I01.9, I02.0, I05-I09.9, I11-I11.9, I20-I25.9, I27.0, I27.2, I28-I28.9, I30-I31.1, I31.8-I37.8, I38-I41.9, I42.1-I42.8, I43-I43.9, I47-I48.9, I51.0-I51.4, I60-I63.9, I65-I66.9, I67.0-I67.3, I67.5-I67.6, I68.0-I68.2, I69.0-I69.3, I70.2-I70.8, I71-I73.9, I77-I83.9, I86-I89.0, I89.9, I98, K75.1
Chronic respiratory diseases	D86-D86.2, D86.9, G47.3, J30-J35.9, J37-J39.9, J41-J46.9, J60-J63.8, J65-J68.9, J70, J70.8-J70.9, J82, J84-J84.9, J91, J91.8-J92.9
Digestive diseases	B18-B18.9, I84-I85.9, I98.2, K20-K20.9, K22-K22.6, K22.8-K29.9, K31-K31.8, K35-K38.9, K40-K42.9, K44-K46.9, K50-K52, K52.2-K52.9, K55-K62, K62.2-K62.6, K62.8-K62.9, K64-K64.9, K66.8, K67, K68, K70-K70.3, K71.7, K73-K75, K75.2, K75.4-K76.2, K76.4-K77, K77.8, K80-K83.9, K85-K86.9, K90-K90.9, K92.8, K93.8, M09.1
Neurological disorders	F00-F02.0, F02.2-F02.3, F02.8-F03.9, G10-G13.8, G20-G20.9, G23-G24, G24.1-G25.0, G25.2-G25.3, G25.5, G25.8-G26.0, G30-G31.1, G31.8-G31.9, G35-G37.9, G40-G41.9, G61-G61.9, G70-G71.1, G71.3-G72, G72.2-G73.7, G90-G90.9, G95-G95.9, M33-M33.9
Mental disorders	F24, F50.0-F50.5
Substance use disorders	E24.4, F10-F16.9, F18-F18.9, G31.2, G62.1, G72.1, P04.3-P04.4, P96.1, Q86.0, R78.0-R78.5, X45-X45.9, X65-X65.9, Y15-Y15.9
Diabetes and kidney diseases	D63.1, E10-E11.9, I12-I13.9, N00-N08.8, N15.0, N18-N18.9, P70.2, Q61-Q62.8
Skin and subcutaneous diseases	A46-A46.0, A66-A67.9, B86, D86.3, I89.1-I89.8, L00-L05.9, L08-L08.9, L10-L14.0, L51-L51.9, L88-L89.9, L97-L98.4, M72.5-M72.6
Musculoskeletal disorders	I27.1, I67.7, L93-L93.2, M00-M03.0, M03.2-M03.6, M05-M09.0, M09.2-M09.8, M30-M32.9, M34-M36.8, M40-M43.1, M65-M65.0, M71.0-M71.1, M80-M82.8, M86.3-M86.4, M87-M87.0, M88-M89.0, M89.5, M89.7-M89.9
Other non-communicable diseases	D25-D26, D28.2, D52.1, D55-D58.9, D59.0-D59.3, D59.5-D59.6, D60-D61.9, D64.0, D66-D67, D68.0-D69.8, D70-D70.2, D70.4-D75.8, D76-D78.8, D86.8, D89-D89.2,

Level 2	ICD-10
	E03-E07.1, E09-E09.9, E15.0, E16.0-E16.9, E20-E24.3, E24.8-E34, E34.1-E34.8, E36-E36.8, E65-E68, E70-E85.2, E88-E89.9, G21.0-G21.1, G24.0, G25.1, G25.4, G25.6-G25.7, G71.2, G72.0, G93.7, G97-G97.9, I95.2-I95.3, I97-I97.9, I98.9, J70.0-J70.5, J95-J95.9, K43-K43.9, K52.0, K62.7, K91-K91.9, K94-K95.8, M87.1, N10-N12.9, N13.6, N14-N15, N15.1-N16.8, N20-N23.0, N25-N28.1, N29-N30.3, N30.8-N32.0, N32.3-N32.4, N34-N34.3, N36-N36.9, N39-N39.2, N41-N41.9, N44-N44.0, N45-N45.9, N49-N49.9, N65-N65.1, N72-N72.0, N75-N77.8, N80-N81.9, N83-N83.9, N99-N99.9, P96.0, P96.2, P96.5, Q00-Q07.9, Q10.4-Q18.9, Q20-Q28.9, Q30-Q36, Q37-Q45.9, Q50-Q60.6, Q63-Q86, Q86.1-Q87.8, Q89-Q89.8, Q90-Q93.9, Q95-Q99.8, R50.2, R95-R95.9
Transport injuries	V00-V86.9, V87.2-V87.3, V88.2-V88.3, V90-V98.8
Unintentional injuries	L55-L55.9, L56.3, L56.8-L56.9, L58-L58.9, N30.4, W00-W46.2, W49-W62.9, W64-W70.9, W73-W75.9, W77-W81.9, W83-W94.9, W97.9, W99-X06.9, X08-X39.9, X47-X48.9, X50-X54.9, X57-X58.9, Y40-Y84.9, Y88-Y88.3
Self-harm and interpersonal violence	U00-U03, X60-X64.9, X66-X83.9, X85-Y08.9, Y35-Y38.9, Y87.0-Y87.1, Y89.0-Y89.1

Appendix Table S4. Mapping of ICD-10 to garbage code

Garbage code hierarchy	ICD-10
Level 1	A40-A41.9, A48.0, A48.3, A49.0-A49.1, A59-A59.9, A71-A71.9, A74.0, B07-B07.9, B30-B30.9, B35-B36.9, B85-B85.4, B87-B88.9, B94.0, D50-D50.0, D50.9, D62-D63.0, D63.8-D64, D64.1-D65.9, D68, D69.9, E15, E16, E50-E50.9, E64.1, E85.3-E87.6, E87.8-E87.9, F06.2-F06.4, F07.2, F09-F09.9, F19-F23.9, F25-F49, F51-F99.0, G06-G08.0, G32-G32.8, G43-G44.2, G44.4-G44.8, G47-G47.2, G47.4-G47.9, G50-G60.9, G62-G62.0, G62.2-G65.2, G80-G83.9, G89-G89.4, G91-G91.2, G91.4-G93, G93.1-G93.2, G93.4-G93.6, G94.0-G94.8, G99-H05, H05.2-H69.9, H71-H99, I26-I26.9, I31.2-I31.4, I46-I46.9, I50.0-I50.4, I76, I95-I95.1, I95.8-I95.9, J69-J69.9, J80-J80.9, J81.0, J85-J85.3, J86-J86.9, J93-J93.1, J93.8-J93.9, J94.2, J96-J96.9, J98.1-J98.3, K00-K19, K30, K65-K66.1, K66.9, K68.1-K68.9, K71-K71.6, K71.8-K72.9, K75.0, L20-L30.9, L40-L50.9, L52-L54.8, L56-L56.2, L56.4-L56.5, L57-L57.9, L59-L68.9, L70-L76.8, L80-L87.9, L90-L92.9, L94-L96, L98.5-L99.8, M04, M10-M12.0, M12.2-M29, M37-M39, M43.2-M49, M49.2-M64, M65.1-M71, M71.2-M72.4, M72.8-M73, M73.8-M79.9, M83-M86.2, M86.5-M86.9, M87.2-M87.9, M89.1-M89.4, M90-M99.9, N17-N17.9, N19-N19.9, N32.1-N32.2, N32.8-N33.8, N35-N35.9, N37-N37.8, N39.3-N39.8, N42-N43.4, N44.1-N44.8, N46-N48.9, N50-N53.9, N61-N64.9, N82-N82.9, N91-N91.5, N95, N95.1-N95.9, N97-N97.9, R02-R02.9, R03.1, R07.0, R08-R09, R09.3, R11-R12.0, R14-R19.6, R19.8-R23, R23.1-R30.9, R32-R50.1, R50.8-R57.9, R58.0-R72.9, R74-R78, R78.6-R94.8, R96-R99.9, U05, U07-U81, U89.9-U99, X40-X44.9, X46-X46.9, X49-X49.9, Y10-Y14.9, Y16-Y19.9, Z00-Z15.8, Z17-unsp.
Level 2	A14.9, A29-A30.9, A45-A45.9, A47-A48, A48.8-A49, A49.3-A49.9, A61-A62, A72-A73, A76, A97, B08-B09, B11-B14, B28-B29, B31-B32.4, B34-B34.9, B61-B62, B68-B68.9, B73-B74.2, B76-B76.9, B78-B81.8, B84, B92-B94, B94.8-B94.9, B95.6-B97.3, B97.7-B99.9, D59, D59.4, D59.8-D59.9, F17-F17.9, G44.3, G91.3, G93.0, G93.3, I10-I10.9, I15-I15.9, I27, I27.8-I27.9, I50, I50.8-I50.9, I67.4, I70-I70.1, I70.9, I74-I75.8, J81, J81.1, J90-J90.0, J94-J94.1, J94.8-J94.9, K92.0-K92.2, N70-N71.9, N73-N74.0, N74.2-N74.8, R03-R03.0, R04-R06.9, R09.0-R09.2, R09.8-R10.9, R13-R13.9, R23.0, R58, S00-T98.3, W47-W48, W63, W71-W72, W76-W76.9, W82, W95-W97, W98, X07, X55-X56, X59-X59.9, Y20-Y34.9, Y86-Y87, Y87.2, Y89, Y89.9-Y99.9

Appendix Table S5A. Decomposition of changes in Health-adjusted life expectancy from 2010 to 2020, by cause and sex

Causes	Males		Females	
	Mortality effects (%)	Disability effects (%)	Mortality effects (%)	Disability effects (%)
HIV/AIDS and sexually transmitted infections	0.01 (0.34)	-0.01 (-0.17)	0.01 (0.55)	-0.01 (-0.53)
Respiratory infections and tuberculosis	-0.10 (-3.69)	0.01 (1.56)	-0.61 (-28.42)	0.01 (9.37)
Enteric infections	0.05 (2.12)	-0.03 (-6.58)	0.03 (1.36)	-0.03 (-45.73)
Neglected tropical diseases and malaria	0.01 (0.09)	-0.02 (-3.47)	-0.01 (-0.02)	-0.03 (-41.43)
Maternal and neonatal disorders	0.00 (0.00)	-0.01 (-1.21)	0.00 (0.00)	-0.01 (-5.73)
Nutritional deficiencies	0.70 (27.32)	-0.11 (-24.02)	1.61 (74.95)	-0.03 (-41.10)
Neoplasms	1.09 (42.11)	0.01 (0.61)	0.37 (17.01)	0.01 (3.99)
Cardiovascular diseases	1.63 (63.13)	0.02 (4.14)	4.45 (206.92)	-0.07 (-96.95)
Chronic respiratory diseases	-3.44 (-133.27)	0.08 (18.21)	-6.20 (-288.49)	0.18 (254.92)
Digestive diseases	0.66 (25.52)	-0.01 (-2.07)	0.33 (15.46)	-0.01 (-3.39)
Neurological disorders	0.37 (14.42)	-0.01 (-3.00)	0.36 (16.97)	-0.02 (-32.84)
Mental disorders	0.04 (1.43)	0.04 (8.76)	-0.01 (-0.17)	0.07 (100.69)
Substance use disorders	-0.03 (-1.15)	0.02 (3.43)	0.00 (0.00)	0.00 (0.00)
Diabetes and kidney diseases	0.05 (2.00)	0.10 (23.41)	-0.24 (-11.14)	0.14 (193.62)
Skin and subcutaneous diseases	-0.15 (-5.84)	0.11 (25.61)	-0.01 (-0.48)	-0.02 (-31.29)
Musculoskeletal disorders	-0.02 (-0.61)	-0.19 (-43.01)	-0.01 (-0.27)	-0.08 (-119.74)
Transport injuries	0.08 (2.93)	0.01 (0.26)	-0.03 (-1.52)	-0.01 (-7.95)
Unintentional injuries	-0.18 (-6.98)	-0.01 (-0.77)	0.01 (0.36)	0.01 (0.87)
Self-harm and interpersonal violence	0.92 (35.83)	-0.13 (-30.32)	1.29 (59.84)	-0.25 (-361.80)
Other infectious diseases	-0.05 (-2.04)	0.01 (0.55)	0.01 (0.39)	0.01 (7.87)
Other non-communicable diseases	0.06 (2.15)	0.01 (2.41)	0.19 (8.77)	-0.05 (-77.85)
Total	1.70 (65.89)	-0.11 (-0.25)	1.55 (72.09)	-0.21 (-3.00)

Appendix Table S5B. Decomposition of changes in Health-adjusted life expectancy from 2020 to 2030, by cause and sex

Causes	Males		Females	
	Mortality effects (%)	Disability effects (%)	Mortality effects (%)	Disability effects (%)
HIV/AIDS and sexually transmitted infections	0.01 (0.37)	0.01 (0.35)	-0.01 (-0.10)	0.01 (0.19)
Respiratory infections and tuberculosis	0.09 (4.06)	0.02 (4.06)	0.14 (6.04)	0.01 (1.75)
Enteric infections	-0.01 (-0.04)	-0.03 (-4.55)	-0.01 (-0.03)	0.01 (0.26)
Neglected tropical diseases and malaria	-0.01 (-0.15)	0.02 (2.95)	-0.01 (-0.03)	0.05 (7.92)
Maternal and neonatal disorders	-0.01 (-0.03)	0.01 (2.46)	-0.01(-0.08)	0.02 (2.91)
Nutritional deficiencies	0.01 (0.42)	-0.01 (-0.33)	-0.41 (-18.11)	-1.88 (-303.74)
Neoplasms	0.17 (8.00)	0.08 (14.30)	0.33 (14.27)	0.04 (7.24)
Cardiovascular diseases	1.06 (49.24)	0.30 (53.83)	2.09 (91.16)	0.58 (93.75)
Chronic respiratory diseases	0.11 (5.12)	0.20 (36.60)	-0.04 (-1.62)	0.08 (12.29)
Digestive diseases	0.09 (4.39)	0.07 (13.38)	0.20 (8.68)	0.10 (16.06)
Neurological disorders	-0.01 (-0.45)	0.02 (3.67)	-0.08 (-3.54)	-0.11 (-18.43)
Mental disorders	-0.01 (-0.03)	0.29 (52.97)	0.01 (0.13)	0.39 (62.16)
Substance use disorders	0.01 (0.17)	0.01 (2.08)	-0.01 (-0.04)	-0.01 (-0.08)
Diabetes and kidney diseases	0.01 (0.18)	0.24 (43.23)	-0.27 (-11.92)	0.15 (23.76)
Skin and subcutaneous diseases	-0.01 (-0.28)	-0.99 (-180.11)	-0.01 (-0.06)	-0.09 (-15.30)
Musculoskeletal disorders	-0.01 (-0.31)	-0.95 (-172.71)	-0.01 (-0.06)	-0.30 (-47.94)
Transport injuries	0.02 (1.00)	0.05 (9.19)	0.02 (0.66)	0.03 (5.39)
Unintentional injuries	0.01 (0.15)	0.01 (1.31)	-0.01 (-0.61)	0.01 (0.74)
Self-harm and interpersonal violence	0.07 (3.42)	0.15 (28.17)	0.04 (1.85)	0.23 (37.66)
Other infectious diseases	0.01 (0.15)	0.01 (0.48)	-0.01 (-0.01)	0.01 (0.30)
Other non-communicable diseases	0.04 (1.17)	0.38 (69.63)	0.05 (1.98)	0.84 (135.39)
Total	1.66 (77.14)	-0.10 (-19.03)	2.02 (88.04)	0.14 (22.27)

Appendix Table S6A. Decomposition of changes in Disability-adjusted life years for males from 2010 to 2020 (95% *UIs*)

Causes	Age structure	Population size	All other reasons
HIV/AIDS and sexually transmitted infections	-170.2 (-174.9, -165.7)	539.7 (536.5, 539.7)	-922.9 (-922.9, -891.0)
Respiratory infections and tuberculosis	1934.3 (1899.9, 1968.2)	2944.5 (2924.9, 2945.0)	-4456.4 (-4552.1, -4300.7)
Enteric infections	653.5 (599.6, 703.7)	900.9 (888.1, 905.9)	3773.2 (3506.7, 3773.2)
Neglected tropical diseases and malaria	434.7 (386.4, 482.9)	631.0 (594.6, 642.9)	3660.0 (3120.5, 3660.0)
Maternal and neonatal disorders	1436.5 (1384.8, 1485.6)	3033.2 (3033.2, 3054.4)	-6046.2 (-6305.5, -6046.2)
Nutritional deficiencies	1553.9 (1312.7, 1793.4)	4754.3 (4645.0, 4948.4)	6698.1 (3674.9, 6698.1)
Neoplasms	31430.3 (31392.4, 31464.3)	45154.4 (45143.7, 45192.3)	-67849.6 (-67849.6, -67644.7)
Cardiovascular diseases	38408.5 (38299.7, 38511.6)	42611.9 (42550.8, 42611.9)	-40181.3 (-41016.6, -39574.2)
Chronic respiratory diseases	15938.9 (15877.3, 16000.0)	14416.2 (14372.2, 14416.2)	-34887.4 (-34887.4, -34036.8)
Digestive diseases	3858.3 (3815.1, 3900.3)	6788.5 (6774.1, 6788.5)	-7748.0 (-7748.0, -7577.9)
Neurological disorders	-642.7 (-858.8, -431.9)	4464.7 (4418.4, 4511.9)	-1839.3 (-3898.4, -257.0)
Mental disorders	3427.2 (3323.2, 3537.0)	3070.3 (3000.7, 3118.5)	716.1 (-988.8, 716.1)
Substance use disorders	-894.5 (-955.4, -835.5)	1954.7 (1903.4, 1955.2)	-2550.2 (-3083.8, -1868.2)
Diabetes and kidney diseases	5937.8 (5819.5, 6073.8)	8858.3 (8682.6, 8942.2)	-3144.9 (-3834.0, -3144.9)
Skin and subcutaneous diseases	3155.4 (2911.7, 3403.5)	6110.8 (5905.8, 6232.8)	12054.7 (12054.7, 14761.4)
Musculoskeletal disorders	2099.2 (1596.3, 2618.6)	16139.6 (15713.6, 16139.6)	41951.6 (40744.5, 49908.3)
Transport injuries	-2445.3 (-2472.3, -2417.3)	5789.6 (5789.6, 5800.7)	-27208.2 (-27336.5, -27208.2)
Unintentional injuries	-1480.0 (-1495.9, -1464.2)	3064.7 (3051.4, 3064.7)	7758.9 (7621.3, 7758.9)
Self-harm and interpersonal violence	3425.3 (3354.3, 3492.8)	6290.9 (6290.7, 6329.6)	558.0 (348.5, 933.5)
Other infectious diseases	269.8 (257.4, 282.4)	783.8 (783.8, 794.6)	-3500.0 (-3500.0, -3488.2)
Other non-communicable diseases	6202.7 (5961.8, 6409.5)	8861.0 (8816.0, 8981.7)	-19522.2 (-19522.2, -17186.6)
Total	114533.6 (112234.8, 116813.0)	187163.0 (185819.1, 187916.7)	-142686.0 (-154374.7, -125015.1)

Appendix Table S6B. Decomposition of changes in Disability-adjusted life years for females from 2010 to 2020 (95% *UIs*)

Causes	Age structure	Population size	All other reasons
HIV/AIDS and sexually transmitted infections	-3.9 (-7.5, -0.4)	166.4 (162.9, 166.4)	-749.1 (-749.1, -740.3)
Respiratory infections and tuberculosis	1129.7 (1100.7, 1157.7)	2007.0 (1999.8, 2010.5)	-3737.7 (-3842.7, -3737.7)
Enteric infections	554.4 (506.8, 604.4)	751.3 (680.8, 751.3)	487.3 (170.6, 611.9)
Neglected tropical diseases and malaria	477.0 (413.8, 539.1)	928.5 (837.7, 928.5)	-1473.1 (-1473.1, -577.0)
Maternal and neonatal disorders	1984.9 (1954.8, 2017.2)	3600.9 (3429.9, 3633.0)	-16885.6 (-16885.6, -15302.5)
Nutritional deficiencies	-94.8 (-689.7, 524.1)	10018.4 (9706.9, 10198.8)	35608.0 (30802.3, 35608.0)
Neoplasms	14568.2 (14548.5, 14592.0)	31643.8 (31643.8, 31669.2)	-31504.0 (-31720.5, -31504.0)
Cardiovascular diseases	27984.9 (27846.9, 28124.2)	38096.9 (38029.8, 38154.8)	-32884.5 (-33627.1, -32884.5)
Chronic respiratory diseases	6126.7 (6007.1, 6250.5)	9022.6 (9018.7, 9037.0)	-28959.2 (-29254.2, -28539.7)
Digestive diseases	2748.7 (2704.7, 2791.7)	4363.2 (4312.0, 4387.2)	-4861.4 (-5065.6, -4777.6)
Neurological disorders	-3237.4 (-3947.5, -2412.6)	6992.0 (6323.6, 7143.6)	18539.0 (14467.6, 18539.0)
Mental disorders	3711.2 (3571.0, 3850.6)	5012.0 (4951.4, 5079.7)	1199.9 (125.7, 1199.9)
Substance use disorders	-510.7 (-577.0, -444.4)	780.7 (734.3, 780.7)	-6777.6 (-6777.6, -6335.9)
Diabetes and kidney diseases	4482.3 (4386.6, 4581.2)	7922.0 (7922.0, 8047.5)	-7732.9 (-8180.3, -7732.9)
Skin and subcutaneous diseases	4897.3 (4593.0, 5243.4)	4489.8 (4338.0, 4656.6)	12701.6 (12701.6, 13880.0)
Musculoskeletal disorders	9724.4 (8858.3, 10541.6)	33376.8 (33291.1, 33657.6)	-9403.8 (-9403.8, -3486.2)
Transport injuries	371.3 (354.3, 389.6)	2893.8 (2893.8, 2930.4)	-8527.7 (-8547.3, -8464.4)
Unintentional injuries	-819.5 (-866.0, -776.8)	2744.7 (2738.0, 2773.5)	3086.3 (2913.1, 3086.3)
Self-harm and interpersonal violence	3203.6 (3097.8, 3315.0)	5655.4 (5616.5, 5747.2)	7231.2 (7150.2, 7900.2)
Other infectious diseases	200.8 (187.3, 213.8)	666.6 (662.6, 666.6)	-1364.6 (-1364.6, -1251.9)
Other non-communicable diseases	4077.3 (3613.3, 4488.4)	23554.3 (23554.3, 24706.0)	-25436.7 (-29706.3, -25436.7)
Total	81576.4 (77657.2, 85590.3)	194687.1 (192847.9, 197126.1)	-101444.6 (-118266.7, -89946.0)

Appendix Table S6C. Decomposition of changes in Disability-adjusted life years for males from 2020 to 2030 (95% *UIs*)

Causes	Age structure	Population size	All other reasons
HIV/AIDS and sexually transmitted infections	110.1 (107.5, 112.8)	550.8 (549.4, 550.8)	813.4 (813.4, 830.8)
Respiratory infections and tuberculosis	2489.1 (2467.2, 2511.4)	2473.9 (2471.6, 2473.9)	-8700.7 (-8704.9, -8665.8)
Enteric infections	2058.5 (2007.2, 2110.1)	2562.0 (2562.0, 2591.9)	12952.0 (12571.9, 12952.0)
Neglected tropical diseases and malaria	624.4 (594.7, 652.4)	963.4 (900.6, 963.4)	-1245.9 (-1246.9, -477.4)
Maternal and neonatal disorders	-210.7 (-242.3, -177.7)	2238.0 (2238.0, 2238.9)	-6386.7 (-6408.8, -6385.8)
Nutritional deficiencies	8330.7 (8102.1, 8559.0)	10958.1 (10876.1, 11042.3)	54410.3 (53309.1, 55484.9)
Neoplasms	41833.8 (41796.2, 41869.6)	40897.4 (40885.5, 40911.8)	-104173.7 (-104360.6, -104015.9)
Cardiovascular diseases	63532.6 (63401.7, 63660.4)	43107.6 (43107.6, 43201.1)	-107730.4 (-108906.3, -107690.2)
Chronic respiratory diseases	28436.3 (28369.2, 28503.1)	14377.5 (14353.0, 14387.0)	-22504.5 (-22644.1, -22183.5)
Digestive diseases	5347.9 (5311.6, 5381.3)	6095.2 (6095.2, 6118.1)	-16854.7 (-17144.2, -16854.7)
Neurological disorders	1365.2 (1136.7, 1601.6)	5706.5 (5682.5, 5798.7)	12007.0 (10978.7, 12209.4)
Mental disorders	1734.7 (1655.7, 1807.5)	2770.5 (2686.0, 2853.0)	-27132.0 (-28004.4, -26236.1)
Substance use disorders	-502.5 (-559.4, -446.9)	1345.7 (1345.7, 1403.2)	-3927.1 (-4599.4, -3927.1)
Diabetes and kidney diseases	9433.8 (9324.0, 9537.4)	9202.0 (9162.3, 9202.0)	-18624.5 (-18624.5, -18104.9)
Skin and subcutaneous diseases	33628.4 (33436.0, 33827.0)	27467.9 (26998.1, 27467.9)	214946.0 (214946.0, 220688.5)
Musculoskeletal disorders	47634.7 (47176.5, 48105.4)	61768.8 (61616.3, 61849.7)	460046.4 (458894.9, 462057.6)
Transport injuries	1145.4 (1118.4, 1169.3)	2866.4 (2855.7, 2883.8)	-8640.7 (-8860.0, -8501.6)
Unintentional injuries	297.2 (284.7, 309.0)	4159.4 (4154.2, 4162.3)	2671.4 (2632.7, 2739.5)
Self-harm and interpersonal violence	6701.9 (6600.5, 6817.4)	8048.4 (8024.7, 8103.3)	2442.0 (1736.6, 2766.3)
Other infectious diseases	1.2 (-3.4, 5.7)	414.8 (414.8, 416.8)	-1233.9 (-1260.1, -1233.9)
Other non-communicable diseases	5471.2 (5277.4, 5662.5)	8249.2 (8249.2, 8489.9)	-6834.2 (-9811.7, -6834.2)
Total	259463.9 (257362.2, 261578.3)	256223.5 (255228.5, 257109.8)	426299.5 (415307.4, 438617.9)

Appendix Table S6D. Decomposition of changes in Disability-adjusted life years for females from 2020 to 2030 (95% *UIs*)

Causes	Age structure	Population size	All other reasons
HIV/AIDS and sexually transmitted infections	64.2 (62.3, 66.2)	105.3 (103.1, 105.5)	288.2 (285.2, 315.2)
Respiratory infections and tuberculosis	1369.8 (1354.6, 1385.2)	1102.5 (1101.0, 1102.5)	-6841.8 (-6841.8, -6818.5)
Enteric infections	1251.6 (1207.9, 1300.0)	1019.5 (1000.3, 1028.5)	2081.8 (1955.6, 2328.0)
Neglected tropical diseases and malaria	1027.0 (970.1, 1085.9)	976.1 (930.9, 1003.8)	1102.8 (748.8, 1666.0)
Maternal and neonatal disorders	-626.1 (-663.1, -592.2)	1692.0 (1671.5, 1703.6)	-96.9 (-222.3, 137.0)
Nutritional deficiencies	57582.2 (57261.9, 57928.9)	75124.7 (74780.8, 75124.7)	726252.2 (726252.2, 730496.0)
Neoplasms	26708.4 (26682.3, 26732.0)	25079.4 (25060.6, 25079.4)	-52177.6 (-52177.6, -51942.5)
Cardiovascular diseases	64800.5 (64552.2, 65049.9)	31504.4 (31401.8, 31583.0)	-107431.4 (-108466.5, -106109.3)
Chronic respiratory diseases	14689.4 (14583.1, 14799.8)	6846.1 (6805.8, 6893.3)	-0.7 (-607.7, 521.7)
Digestive diseases	5190.7 (5137.8, 5243.2)	3294.9 (3277.7, 3303.2)	-11176.9 (-11295.5, -10951.4)
Neurological disorders	5085.2 (4580.6, 5625.8)	12157.4 (12151.9, 12411.4)	56729.5 (53482.8, 56982.8)
Mental disorders	2953.8 (2831.4, 3073.6)	3681.9 (3678.2, 3976.5)	-37066.7 (-40036.7, -36983.6)
Substance use disorders	5.6 (5.3, 5.9)	36.0 (35.9, 36.2)	407.3 (405.7, 409.5)
Diabetes and kidney diseases	13103.0 (12998.0, 13207.0)	9000.6 (8987.9, 9041.9)	14562.7 (14028.6, 14754.4)
Skin and subcutaneous diseases	15253.7 (15066.0, 15435.2)	9335.8 (9155.0, 9364.8)	31401.2 (30985.3, 33639.1)
Musculoskeletal disorders	41738.8 (40994.2, 42432.4)	62785.9 (62128.0, 63636.6)	365745.2 (355286.1, 373912.3)
Transport injuries	889.1 (868.5, 910.6)	1599.9 (1599.9, 1602.7)	-3516.1 (-3548.3, -3516.1)
Unintentional injuries	364.7 (323.5, 405.4)	2696.9 (2696.9, 2712.4)	107.5 (-63.3, 107.5)
Self-harm and interpersonal violence	10481.5 (10293.9, 10679.0)	7594.8 (7523.3, 7652.0)	6269.7 (5510.0, 7197.2)
Other infectious diseases	113.0 (102.5, 123.5)	429.6 (427.2, 434.2)	-597.0 (-655.3, -563.7)
Other non-communicable diseases	10170.1 (9646.0, 10683.0)	16428.5 (16428.5, 16894.7)	-51714.9 (-57495.7, -51714.9)
Total	272216.2 (268859.0, 275580.3)	272492.2 (270946.2, 274690.9)	934328.1 (907529.6, 953866.7)

Appendix Table S7. The absolute number and percentage of changes in the disease burden in 2030 compared to 2020 (95% *UIs*)

	Scenario 1		Scenario 2	
	Absolute number (person-years)	Percentage (%)	Absolute number (person-years)	Percentage (%)
Mortality burden				
Males	319625.7 (280663.5, 360776.0)	45.0 (39.5, 50.8)	-20977.2 (-49699.1, 9578.0)	-2.9 (-7.0, 1.3)
Females	234507.8 (213316.0, 255112.9)	54.7 (49.8, 59.5)	-5598.5 (-20901.8, 9709.4)	-1.3 (-4.9, 2.3)
Disability burden				
Males	127506.8 (103455.9, 152860.2)	31.8 (25.8, 38.2)	50818.6 (30539.9, 72260.1)	12.7 (7.6, 18.0)
Females	178062.0 (149258.9, 207560.0)	33.8 (28.3, 39.4)	77367.6 (53714.5, 101388.0)	14.7 (10.2, 19.2)

Appendix Table S8A. In Scenario 1, the absolute number and percentage of increase in the disease burden for males in 2030 compared to 2020, by cause (95% *UIs*)

Causes	Mortality burden		Disability burden	
	Absolute number (person-years)	Percentage (%)	Absolute number (person-years)	Percentage (%)
HIV/AIDS and sexually transmitted infections	458.5 (308.8, 617.4)	20.4 (13.7, 27.4)	83.2 (58.7, 109.1)	21.8 (15.4, 28.6)
Respiratory infections and tuberculosis	5705.3 (4923.2, 6540.8)	47.0 (40.5, 53.8)	1045.5 (763.4, 1341.8)	26.0 (19.0, 33.3)
Enteric infections	323.2 (164.8, 495.9)	23.4 (11.9, 35.9)	1927.2 (1418.4, 2464.6)	30.8 (22.7, 39.4)
Neglected tropical diseases and malaria	99.3 (81.5, 118.1)	27.8 (22.8, 33.0)	1545.6 (1285.0, 1819.6)	29.1 (24.2, 34.3)
Maternal and neonatal disorders	1024.8 (-536.8, 2828.1)	18.0 (-9.4, 49.7)	1368.7 (548.5, 2238.2)	13.9 (5.6, 22.7)
Nutritional deficiencies	2698.2 (2490.2, 2913.5)	63.7 (58.8, 68.8)	11950.6 (10475.1, 13504.2)	42.5 (37.3, 48.0)
Neoplasms	99418.7 (87300.0, 112122.4)	40.5 (35.6, 45.7)	2734.8 (2425.7, 3058.4)	43.1 (38.2, 48.2)
Cardiovascular diseases	120453.9 (109073.0, 132319.1)	51.4 (46.5, 56.4)	9630.3 (8657.5, 10645.3)	47.2 (42.5, 52.2)
Chronic respiratory diseases	42737.7 (39850.6, 45726.6)	66.8 (62.3, 71.5)	6177.0 (5660.1, 6714.9)	53.6 (49.1, 58.3)
Digestive diseases	11750.3 (10026.7, 13572.2)	39.1 (33.4, 45.2)	2694.1 (2177.1, 3241.7)	30.8 (24.9, 37.0)
Neurological disorders	2541.0 (2026.8, 3095.0)	36.9 (29.4, 44.9)	2783.5 (1343.6, 4315.3)	14.8 (7.2, 23.0)
Mental disorders	0.0 (0.0, 0.0)	0.0 (0.0, 0.0)	8986.8 (7768.9, 10269.3)	37.9 (32.7, 43.3)
Substance use disorders	403.1 (217.3, 600.1)	16.0 (8.6, 23.9)	584.5 (31.5, 1172.4)	8.0 (0.4, 16.0)
Diabetes and kidney diseases	12348.7 (10920.5, 13848.8)	44.0 (38.9, 49.3)	9128.6 (7726.2, 10602.9)	34.7 (29.3, 40.3)
Skin and subcutaneous diseases	389.4 (341.3, 439.8)	41.6 (36.5, 47.0)	11082.3 (8454.7, 13853.1)	25.4 (19.4, 31.7)
Musculoskeletal disorders	592.3 (471.1, 724.7)	39.7 (31.6, 48.6)	34352.2 (26530.5, 42603.7)	28.6 (22.1, 35.5)
Transport injuries	3445.2 (2476.4, 4471.4)	23.3 (16.7, 30.2)	1722.2 (1496.6, 1959.0)	39.2 (34.1, 44.6)
Unintentional injuries	3835.9 (2533.2, 5227.0)	20.5 (13.6, 28.0)	630.1 (444.1, 827.3)	22.2 (15.7, 29.2)
Self-harm and interpersonal violence	8606.3 (6487.5, 10890.2)	32.4 (24.4, 41.0)	5992.6 (5282.7, 6736.5)	44.2 (39.0, 49.7)
Other infectious diseases	401.4 (209.5, 614.3)	21.1 (11.0, 32.3)	119.6 (54.4, 189.1)	12.3 (5.6, 19.4)
Other non-communicable diseases	2392.3 (1297.9, 3610.6)	26.2 (14.2, 39.5)	12967.4 (10853.2, 15193.8)	34.4 (28.8, 40.3)

Causes	Mortality burden		Disability burden	
	Absolute number (person-years)	Percentage (%)	Absolute number (person-years)	Percentage (%)
Total	319625.7 (280663.5, 360776.0)	45.0 (39.5, 50.8)	127506.8 (103455.9, 152860.2)	31.8 (25.8, 38.2)

Appendix Table S8B. In Scenario 1, the absolute number and percentage of increase in the disease burden for females in 2030 compared to 2020, by cause (95% *UIs*)

Causes	Mortality burden		Disability burden	
	Absolute number (person-years)	Percentage (%)	Absolute number (person-years)	Percentage (%)
HIV/AIDS and sexually transmitted infections	97.5 (81.3, 114.2)	37.7 (31.5, 44.2)	61.5 (52.2, 71.0)	41.9 (35.5, 48.3)
Respiratory infections and tuberculosis	3478.4 (3179.4, 3762.9)	60.2 (55.1, 65.2)	512.6 (352.2, 680.7)	21.0 (14.5, 27.9)
Enteric infections	309.3 (212.9, 412.7)	39.8 (27.4, 53.1)	1263.2 (1029.2, 1497.5)	39.3 (32.0, 46.6)
Neglected tropical diseases and malaria	79.3 (74.4, 84.0)	48.6 (45.7, 51.5)	1936.0 (1816.8, 2052.1)	48.4 (45.4, 51.3)
Maternal and neonatal disorders	876.4 (-165.7, 2076.7)	22.8 (-4.3, 54.1)	253.4 (-206.1, 731.4)	4.5 (-3.6, 12.9)
Nutritional deficiencies	4451.0 (4152.6, 4702.7)	69.6 (64.9, 73.5)	15740.2 (11518.8, 20117.9)	25.5 (18.7, 32.6)
Neoplasms	59534.1 (52726.3, 66425.2)	41.8 (37.0, 46.6)	2025.7 (1765.9, 2290.4)	39.2 (34.2, 44.3)
Cardiovascular diseases	109967.0 (103605.9, 115572.2)	68.5 (64.6, 72.0)	15802.1 (14777.9, 16749.7)	61.8 (57.8, 65.6)
Chronic respiratory diseases	15622.1 (14794.8, 16316.1)	74.3 (70.4, 77.6)	6288.2 (5846.9, 6712.0)	64.7 (60.1, 69.0)
Digestive diseases	8200.0 (7697.0, 8651.2)	64.3 (60.4, 67.9)	3308.3 (2949.4, 3666.4)	44.0 (39.2, 48.7)
Neurological disorders	3214.2 (2834.0, 3599.4)	50.0 (44.1, 56.0)	8159.0 (5885.2, 10560.5)	24.8 (17.9, 32.0)
Mental disorders	62.6 (60.5, 64.2)	115.4 (111.7, 118.5)	13750.0 (12329.1, 15192.7)	43.5 (39.0, 48.0)
Substance use disorders	0.0 (0.0, 0.0)	0.0 (0.0, 0.0)	4.7 (4.1, 5.4)	50.2 (43.9, 56.9)
Diabetes and kidney diseases	10808.6 (9991.7, 11594.6)	56.2 (51.9, 60.3)	7937.7 (7093.0, 8780.4)	43.4 (38.8, 48.0)
Skin and subcutaneous diseases	500.9 (471.8, 526.6)	63.2 (59.5, 66.4)	12897.4 (11753.4, 14041.5)	41.2 (37.5, 44.8)
Musculoskeletal disorders	854.3 (678.7, 1033.7)	27.4 (21.8, 33.1)	45036.2 (36065.0, 54312.9)	27.6 (22.1, 33.2)
Transport injuries	1464.7 (1035.5, 1915.0)	23.3 (16.5, 30.5)	1504.4 (1329.0, 1682.2)	40.6 (35.9, 45.4)
Unintentional injuries	2342.6 (1715.4, 2998.9)	23.6 (17.3, 30.3)	796.6 (466.7, 1144.4)	16.9 (9.9, 24.3)
Self-harm and interpersonal violence	8683.6 (7441.9, 9941.8)	48.0 (41.1, 55.0)	8538.5 (7813.8, 9225.4)	53.9 (49.3, 58.2)
Other infectious diseases	393.1 (260.9, 535.1)	29.8 (19.7, 40.5)	269.9 (183.1, 360.8)	21.2 (14.4, 28.3)
Other non-communicable diseases	3568.3 (2466.7, 4785.7)	38.9 (26.9, 52.2)	31976.4 (26433.3, 37684.7)	32.5 (26.8, 38.3)

Causes	Mortality burden		Disability burden	
	Absolute number (person-years)	Percentage (%)	Absolute number (person-years)	Percentage (%)
Total	234507.8 (213316.0, 255112.9)	54.7 (49.8, 59.5)	178062.0 (149258.9, 207560.0)	33.8 (28.3, 39.4)

Appendix Table S8C. In Scenario 2, the absolute number and percentage of increase in the disease burden for males in 2030 compared to 2020, by cause (95% *UIs*)

Causes	Mortality burden		Disability burden	
	Absolute number (person-years)	Percentage (%)	Absolute number (person-years)	Percentage (%)
HIV/AIDS and sexually transmitted infections	1493.5 (1280.3, 1719.7)	66.3 (56.8, 76.3)	-12.8 (-32.6, 8.1)	-3.3 (-8.5, 2.1)
Respiratory infections and tuberculosis	-3094.2 (-3574.0, -2574.3)	-25.5 (-29.4, -21.2)	-615.1 (-807.5, -411.3)	-15.3 (-20.1, -10.2)
Enteric infections	389.5 (188.3, 613.3)	28.2 (13.6, 44.4)	17228.7 (15935.8, 18614.5)	275.2 (254.6, 297.4)
Neglected tropical diseases and malaria	121.1 (96.7, 147.0)	33.9 (27.1, 41.1)	183.9 (-50.4, 430.7)	3.5 (-0.9, 8.1)
Maternal and neonatal disorders	4587.5 (2234.3, 7304.1)	80.7 (39.3, 128.5)	-9023.2 (-9143.4, -8889.3)	-91.5 (-92.7, -90.1)
Nutritional deficiencies	2009.3 (1624.7, 2424.7)	47.4 (38.4, 57.3)	71293.2 (65877.9, 77062.0)	253.5 (234.3, 274.0)
Neoplasms	-24543.1 (-32723.9, -15953.7)	-10.0 (-13.3, -6.5)	3049.6 (2709.2, 3406.8)	48.1 (42.7, 53.7)
Cardiovascular diseases	-10357.9 (-17480.4, -2924.6)	-4.4 (-7.5, -1.2)	8740.5 (7798.4, 9724.7)	42.9 (38.3, 47.7)
Chronic respiratory diseases	14665.2 (12569.3, 16830.7)	22.9 (19.6, 26.3)	5652.6 (5121.3, 6206.6)	49.1 (44.4, 53.9)
Digestive diseases	-6611.4 (-7609.2, -5557.9)	-22.0 (-25.3, -18.5)	946.4 (484.7, 1434.4)	10.8 (5.5, 16.4)
Neurological disorders	3960.1 (3412.5, 4546.2)	57.5 (49.6, 66.0)	15151.0 (13070.2, 17358.6)	80.8 (69.7, 92.5)
Mental disorders	503.2 (475.8, 532.3)	100.0 (100.0, 100.0)	-23152.3 (-23189.0, -23113.2)	-97.6 (-97.7, -97.4)
Substance use disorders	-848.9 (-951.9, -739.8)	-33.8 (-37.9, -29.4)	-2507.9 (-2833.0, -2162.8)	-34.3 (-38.7, -29.6)
Diabetes and kidney diseases	-861.6 (-1781.2, 100.4)	-3.1 (-6.3, 0.4)	1178.4 (110.6, 2300.3)	4.5 (0.4, 8.7)
Skin and subcutaneous diseases	-800.7 (-806.3, -794.7)	-85.5 (-86.1, -84.9)	-11397.3 (-12344.1, -10409.8)	-26.1 (-28.3, -23.8)
Musculoskeletal disorders	-1221.6 (-1233.0, -1209.5)	-81.9 (-82.6, -81.1)	-51366.3 (-53971.8, -48630.6)	-42.8 (-44.9, -40.5)
Transport injuries	-6039.9 (-6552.7, -5494.6)	-40.8 (-44.3, -37.1)	1385.9 (1145.4, 1639.3)	31.6 (26.1, 37.3)
Unintentional injuries	5864.3 (4419.9, 7396.2)	31.4 (23.7, 39.6)	1343.6 (1094.2, 1607.8)	47.4 (38.6, 56.7)
Self-harm and interpersonal violence	-1395.3 (-2986.0, 315.6)	-5.2 (-11.2, 1.2)	18770.1 (17516.8, 20085.1)	138.5 (129.3, 148.2)
Other infectious diseases	-572.8 (-686.0, -449.3)	-30.1 (-36.1, -23.6)	-259.6 (-303.7, -212.8)	-26.6 (-31.2, -21.8)
Other non-communicable diseases	1776.5 (383.7, 3346.2)	19.5 (4.2, 36.6)	4229.0 (2350.9, 6211.0)	11.2 (6.2, 16.5)

Causes	Mortality burden		Disability burden	
	Absolute number (person-years)	Percentage (%)	Absolute number (person-years)	Percentage (%)
Total	-20977.2 (-49699.1, 9578.0)	-2.9 (-7.0, 1.3)	50818.6 (30539.9, 72260.1)	12.7 (7.6, 18.0)

Appendix Table S8D. In Scenario 2, the absolute number and percentage of increase in the disease burden for females in 2030 compared to 2020, by cause (95% *UIs*)

Causes	Mortality burden		Disability burden	
	Absolute number (person-years)	Percentage (%)	Absolute number (person-years)	Percentage (%)
HIV/AIDS and sexually transmitted infections	340.6 (309.7, 372.7)	131.8 (119.9, 144.3)	124.5 (109.8, 139.7)	84.7 (74.7, 95.0)
Respiratory infections and tuberculosis	-3273.8 (-3406.6, -3135.9)	-56.7 (-59.0, -54.3)	-1039.4 (-1120.8, -954.0)	-42.7 (-46.0, -39.2)
Enteric infections	98.0 (29.9, 171.2)	12.6 (3.8, 22.0)	4474.5 (4180.5, 4763.2)	139.3 (130.2, 148.3)
Neglected tropical diseases and malaria	279.6 (257.3, 302.8)	171.6 (157.9, 185.8)	3006.6 (2723.9, 3294.9)	75.2 (68.1, 82.4)
Maternal and neonatal disorders	3452.4 (1827.5, 5324.9)	89.9 (47.6, 138.6)	-2383.0 (-2748.1, -1989.8)	-42.0 (-48.5, -35.1)
Nutritional deficiencies	-4373.0 (-4443.2, -4307.8)	-68.4 (-69.5, -67.4)	29142.5 (25128.2, 33328.4)	47.2 (40.7, 54.0)
Neoplasms	-2807.0 (-7680.0, 2146.9)	-2.0 (-5.4, 1.5)	2494.2 (2206.8, 2787.8)	48.2 (42.7, 53.9)
Cardiovascular diseases	-21693.5 (-24999.2, -18737.7)	-13.5 (-15.6, -11.7)	10776.2 (9842.8, 11652.7)	42.2 (38.5, 45.6)
Chronic respiratory diseases	7308.6 (6683.2, 7829.6)	34.8 (31.8, 37.2)	14243.9 (13634.8, 14803.3)	146.5 (140.2, 152.2)
Digestive diseases	-3621.9 (-3891.0, -3365.6)	-28.4 (-30.5, -26.4)	861.6 (542.1, 1185.9)	11.4 (7.2, 15.8)
Neurological disorders	5501.5 (4990.2, 6021.5)	85.5 (77.6, 93.6)	67927.8 (62498.6, 73593.6)	206.1 (189.7, 223.3)
Mental disorders	146.6 (135.8, 157.6)	270.5 (250.6, 290.9)	-31496.7 (-31505.7, -31487.3)	-99.6 (-99.6, -99.5)
Substance use disorders	154.9 (145.3, 164.8)	100.0 (100.0, 100.0)	294.4 (276.1, 313.6)	3122.7 (2928.3, 3326.5)
Diabetes and kidney diseases	17950.2 (16988.7, 18866.6)	93.3 (88.3, 98.1)	18643.1 (17512.9, 19759.1)	101.9 (95.7, 108.0)
Skin and subcutaneous diseases	288.0 (255.6, 318.4)	36.3 (32.2, 40.2)	56188.7 (54351.1, 57876.9)	179.4 (173.6, 184.8)
Musculoskeletal disorders	-2644.4 (-2666.1, -2622.0)	-84.8 (-85.5, -84.1)	-100195.6 (-102728.3, -97597.5)	-61.3 (-62.8, -59.7)
Transport injuries	-2760.4 (-2974.0, -2536.0)	-43.9 (-47.3, -40.4)	1688.9 (1483.9, 1898.0)	45.6 (40.1, 51.3)
Unintentional injuries	980.2 (421.8, 1564.4)	9.9 (4.3, 15.8)	2139.7 (1726.6, 2575.0)	45.4 (36.6, 54.6)
Self-harm and interpersonal violence	949.5 (-22.6, 1943.1)	5.3 (-0.1, 10.7)	23590.8 (22340.1, 24802.4)	148.9 (141.0, 156.5)
Other infectious diseases	20.6 (-100.3, 152.6)	1.6 (-7.6, 11.5)	-108.0 (-173.5, -39.4)	-8.5 (-13.6, -3.1)
Other non-communicable diseases	-1895.4 (-2763.8, -922.7)	-20.7 (-30.2, -10.1)	-23007.2 (-26567.3, -19318.5)	-23.4 (-27.0, -19.6)

Causes	Mortality burden		Disability burden	
	Absolute number (person-years)	Percentage (%)	Absolute number (person-years)	Percentage (%)
Total	-5598.5 (-20901.8, 9709.4)	-1.3 (-4.9, 2.3)	77367.6 (53714.5, 101388.0)	14.7 (10.2, 19.2)

Appendix Table S9A. Expected burden of disease for males in 2030 and the reduction compared to 2020, by cause (95% *UIs*)

Causes	Mortality burden (person-years per 100,000 people)		Disability burden (person-years per 100,000 people)	
	Expectation	Reduction	Expectation	Reduction
HIV/AIDS and sexually transmitted infections	39.0 (36.4, 41.8)	7.0 (4.2, 9.6)	6.6 (6.2, 7.1)	1.2 (0.7, 1.6)
Respiratory infections and tuberculosis	210.4 (196.4, 225.5)	38.0 (22.9, 52.0)	69.7 (65.1, 74.7)	12.6 (7.6, 17.2)
Enteric infections	23.9 (22.3, 25.6)	4.3 (2.6, 5.9)	108.4 (101.2, 116.1)	19.6 (11.8, 26.8)
Neglected tropical diseases and malaria	6.2 (5.8, 6.6)	1.1 (0.7, 1.5)	92.0 (85.9, 98.6)	16.6 (10.0, 22.7)
Maternal and neonatal disorders	98.4 (91.9, 105.5)	17.8 (10.7, 24.3)	170.7 (159.4, 183.0)	30.8 (18.6, 42.2)
Nutritional deficiencies	73.3 (68.4, 78.6)	13.2 (8.0, 18.1)	486.8 (454.5, 521.7)	87.9 (53.0, 120.3)
Neoplasms	4246.4 (3964.4, 4551.2)	767.1 (462.3, 1049.1)	109.8 (102.5, 117.7)	19.8 (12.0, 27.1)
Cardiovascular diseases	4059.5 (3789.9, 4350.9)	733.4 (442.0, 1002.9)	352.9 (329.5, 378.3)	63.8 (38.4, 87.2)
Chronic respiratory diseases	1107.9 (1034.3, 1187.4)	200.1 (120.6, 273.7)	199.5 (186.2, 213.8)	36.0 (21.7, 49.3)
Digestive diseases	519.8 (485.3, 557.1)	93.9 (56.6, 128.4)	151.5 (141.5, 162.4)	27.4 (16.5, 37.4)
Neurological disorders	119.2 (111.3, 127.8)	21.5 (13.0, 29.5)	324.7 (303.2, 348.0)	58.7 (35.4, 80.2)
Mental disorders	0.0 (0.0, 0.0)	0.0 (0.0, 0.0)	410.8 (383.5, 440.3)	88.8 (59.3, 116.1)
Substance use disorders	43.5 (40.6, 46.6)	7.9 (4.7, 10.8)	126.6 (118.2, 135.6)	22.9 (13.8, 31.3)
Diabetes and kidney diseases	486.2 (453.9, 521.1)	87.8 (52.9, 120.1)	455.9 (425.6, 488.6)	82.4 (49.6, 112.6)
Skin and subcutaneous diseases	16.2 (15.1, 17.4)	2.9 (1.8, 4.0)	755.7 (705.5, 809.9)	136.5 (82.3, 186.7)
Musculoskeletal disorders	25.8 (24.1, 27.7)	4.7 (2.8, 6.4)	2079.1 (1941.1, 2228.4)	375.6 (226.4, 513.7)
Transport injuries	256.2 (239.2, 274.6)	46.3 (27.9, 63.3)	76.0 (71.0, 81.5)	13.7 (8.3, 18.8)
Unintentional injuries	323.2 (301.7, 346.4)	58.4 (35.2, 79.8)	49.1 (45.8, 52.6)	8.9 (5.3, 12.1)
Self-harm and interpersonal violence	460.2 (429.7, 493.3)	83.1 (50.1, 113.7)	234.5 (219.0, 251.4)	42.4 (25.5, 57.9)
Other infectious diseases	32.9 (30.7, 35.3)	5.9 (3.6, 8.1)	16.9 (15.8, 18.1)	3.0 (1.8, 4.2)
Other non-communicable diseases	158.1 (147.6, 169.4)	28.6 (17.2, 39.1)	653.5 (610.1, 700.4)	118.1 (71.1, 161.4)

Causes	Mortality burden (person-years per 100,000 people)		Disability burden (person-years per 100,000 people)	
	Expectation	Reduction	Expectation	Reduction
Total	12306.3 (11489.0, 13189.8)	2223.2 (1339.8, 3040.3)	6930.71 (6470.8, 7428.2)	1266.69 (769.1, 1726.8)

Appendix Table S9B. Expected burden of disease for females in 2030 and the reduction compared to 2020, by cause (95% *UIs*)

Causes	Mortality burden (person-years per 100,000 people)		Disability burden (person-years per 100,000 people)	
	Expectation	Reduction	Expectation	Reduction
HIV/AIDS and sexually transmitted infections	4.4 (4.1, 4.7)	0.8 (0.5, 1.1)	2.5 (2.3, 2.7)	0.5 (0.3, 0.6)
Respiratory infections and tuberculosis	98.5 (92.3, 105.3)	17.9 (11.1, 24.2)	41.6 (38.9, 44.4)	7.6 (4.7, 10.2)
Enteric infections	13.3 (12.4, 14.2)	2.4 (1.5, 3.3)	54.8 (51.3, 58.6)	10.0 (6.2, 13.5)
Neglected tropical diseases and malaria	2.8 (2.6, 3.0)	0.5 (0.3, 0.7)	68.3 (63.9, 73.0)	12.4 (7.7, 16.8)
Maternal and neonatal disorders	65.5 (61.4, 70.1)	11.9 (7.4, 16.1)	96.8 (90.6, 103.4)	17.6 (10.9, 23.8)
Nutritional deficiencies	109.1 (102.2, 116.6)	19.9 (12.3, 26.8)	1053.1 (986.2, 1125.7)	191.6 (119.0, 258.6)
Neoplasms	2432.5 (2277.8, 2600.1)	442.6 (274.9, 597.2)	88.2 (82.6, 94.3)	16.0 (10.0, 21.7)
Cardiovascular diseases	2738.3 (2564.2, 2927.0)	498.2 (309.5, 672.3)	436.0 (408.2, 466.0)	79.3 (49.3, 107.0)
Chronic respiratory diseases	358.7 (335.9, 383.4)	65.3 (40.5, 88.1)	165.9 (155.4, 177.3)	30.2 (18.7, 40.7)
Digestive diseases	217.5 (203.7, 232.5)	39.6 (24.6, 53.4)	128.4 (120.2, 137.3)	23.4 (14.5, 31.5)
Neurological disorders	109.8 (102.8, 117.3)	20.0 (12.4, 26.9)	562.3 (526.5, 601.0)	102.3 (63.5, 138.0)
Mental disorders	0.9 (0.9, 1.0)	0.2 (0.1, 0.3)	539.7 (505.4, 576.9)	121.0 (83.8, 155.3)
Substance use disorders	0.0 (0.0, 0.0)	0.0 (0.0, 0.0)	0.2 (0.2, 0.2)	0.0 (0.0, 0.0)
Diabetes and kidney diseases	328.2 (307.4, 350.9)	59.7 (37.1, 80.6)	312.3 (292.4, 333.8)	56.8 (35.3, 76.7)
Skin and subcutaneous diseases	13.5 (12.7, 14.5)	2.5 (1.5, 3.3)	534.3 (500.3, 571.1)	97.2 (60.4, 131.2)
Musculoskeletal disorders	53.2 (49.8, 56.9)	9.7 (6.0, 13.1)	2789.0 (2611.7, 2981.3)	507.4 (315.2, 684.7)
Transport injuries	107.2 (100.4, 114.6)	19.5 (12.1, 26.3)	63.2 (59.1, 67.5)	11.5 (7.1, 15.5)
Unintentional injuries	169.1 (158.4, 180.8)	30.8 (19.1, 41.5)	80.5 (75.4, 86.0)	14.6 (9.1, 19.8)
Self-harm and interpersonal violence	308.6 (289.0, 329.9)	56.1 (34.9, 75.8)	270.3 (253.2, 289.0)	49.2 (30.6, 66.4)
Other infectious diseases	22.5 (21.1, 24.1)	4.1 (2.5, 5.5)	21.8 (20.4, 23.3)	4.0 (2.5, 5.3)
Other non-communicable diseases	156.4 (146.4, 167.2)	28.5 (17.7, 38.4)	1680.2 (1573.4, 1796.0)	305.7 (189.9, 412.5)

Causes	Mortality burden (person-years per 100,000 people)		Disability burden (person-years per 100,000 people)	
	Expectation	Reduction	Expectation	Reduction
Total	7310.1 (6845.5, 7814.1)	1330.0 (826.0, 1794.9)	8989.3 (8417.6, 9608.8)	8989.3 (1038.7, 2229.8)
